# Supplementary material for: Altered milk tryptophan and tryptophan metabolites in women living with HIV
Source: Nat Commun. 2025 Oct 28;16:9437. doi: 10.1038/s41467-025-64566-w (PMC12568960; doi:10.1038/s41467-025-64566-w)
Supplement: Supplementary file 1 — Supplementary Information [file 41467_2025_64566_MOESM1_ESM.pdf]

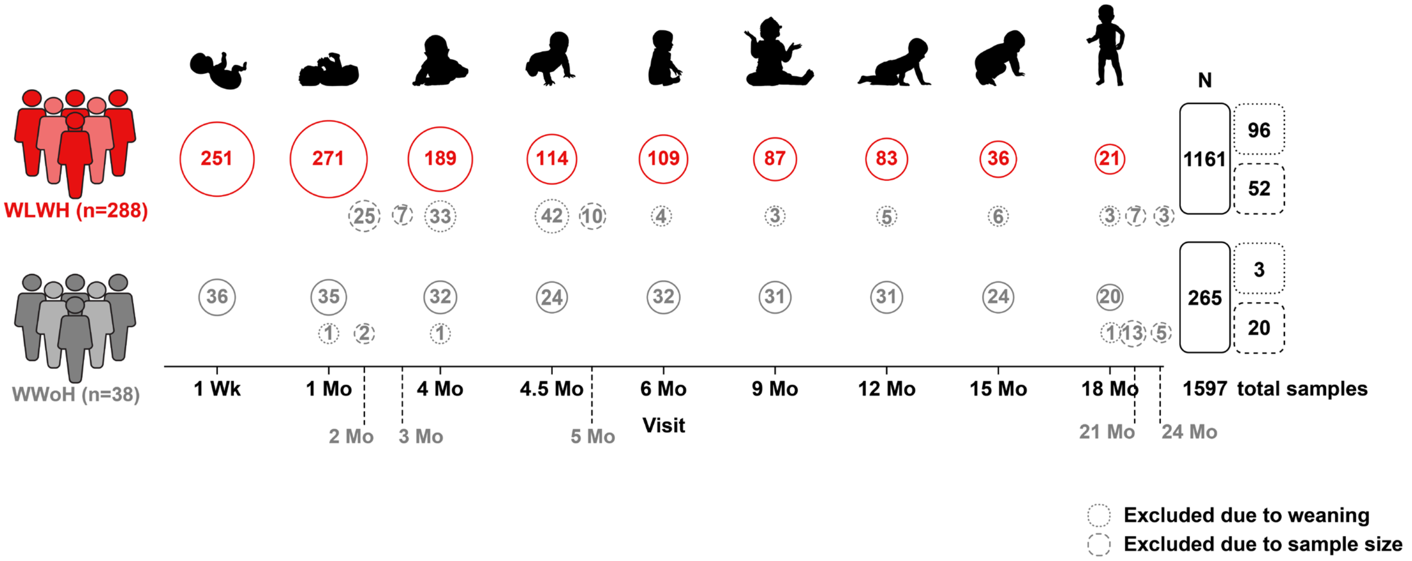


**Supplementary Figure 1**

Study design showing samples included and excluded in the final analysis. Of the 1599 milk samples, 1597 were successfully characterized. Samples were later excluded either because they were collected post-weaning (N=96 samples in WLWH and 3 samples in WWoH) or there were not enough samples at a given timepoint for comparison (N= 52 samples in WLWH and 20 samples in WWoH). Open circles denote number of samples at each study visit included in the final analysis. The number of participants in each group is indicated in parentheses on the left, and the number of samples is indicated along the right. Dotted circles denote samples excluded from the final analysis due to weaning. Dashed circles denote samples excluded from the final analysis due to insufficient sample size at the indicated timepoint.

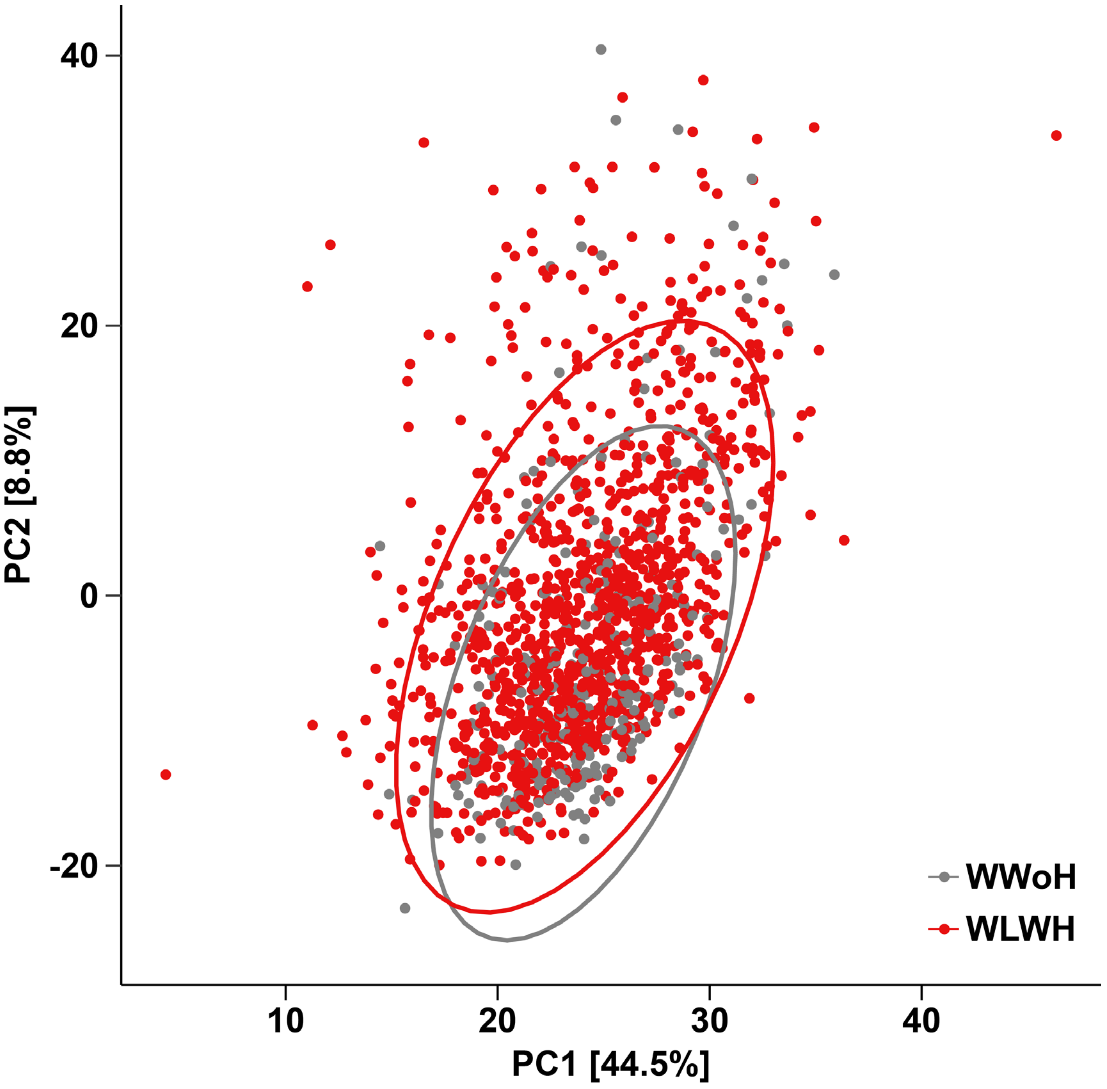


**Supplementary Figure 2**
Principal coordinates analysis plot of milk metabolomic profiles colored by maternal HIV infection. Ellipses show 95% confidence areas for the groups as marked. Numbers in brackets denote percent of overall variation explained by each component.

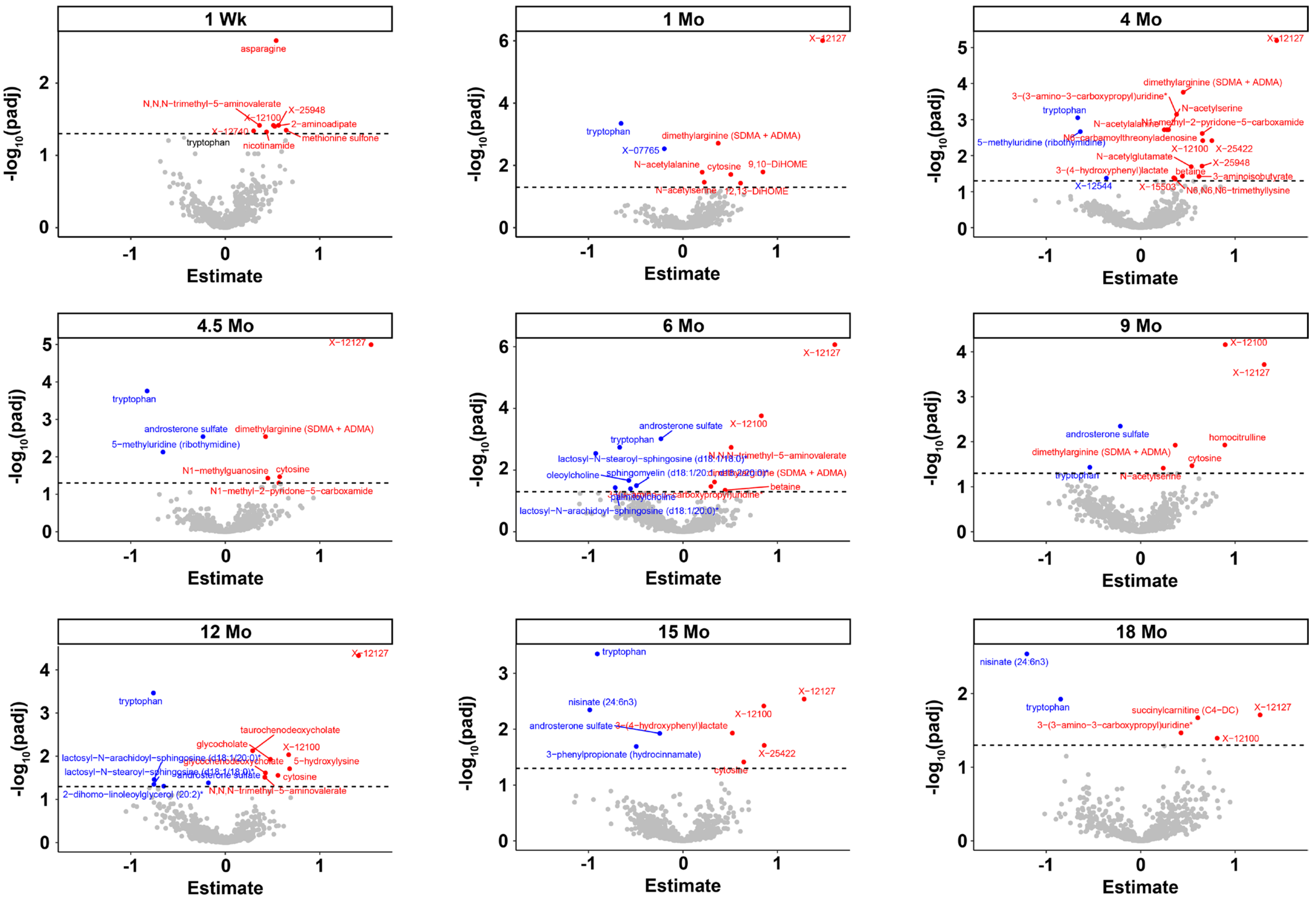


**Supplementary Figure 3**

Volcano plots of differences in milk metabolite abundances at each study timepoint. Blue and red points are significantly decreased and increased metabolites in WLWH versus WWoH, respectively.

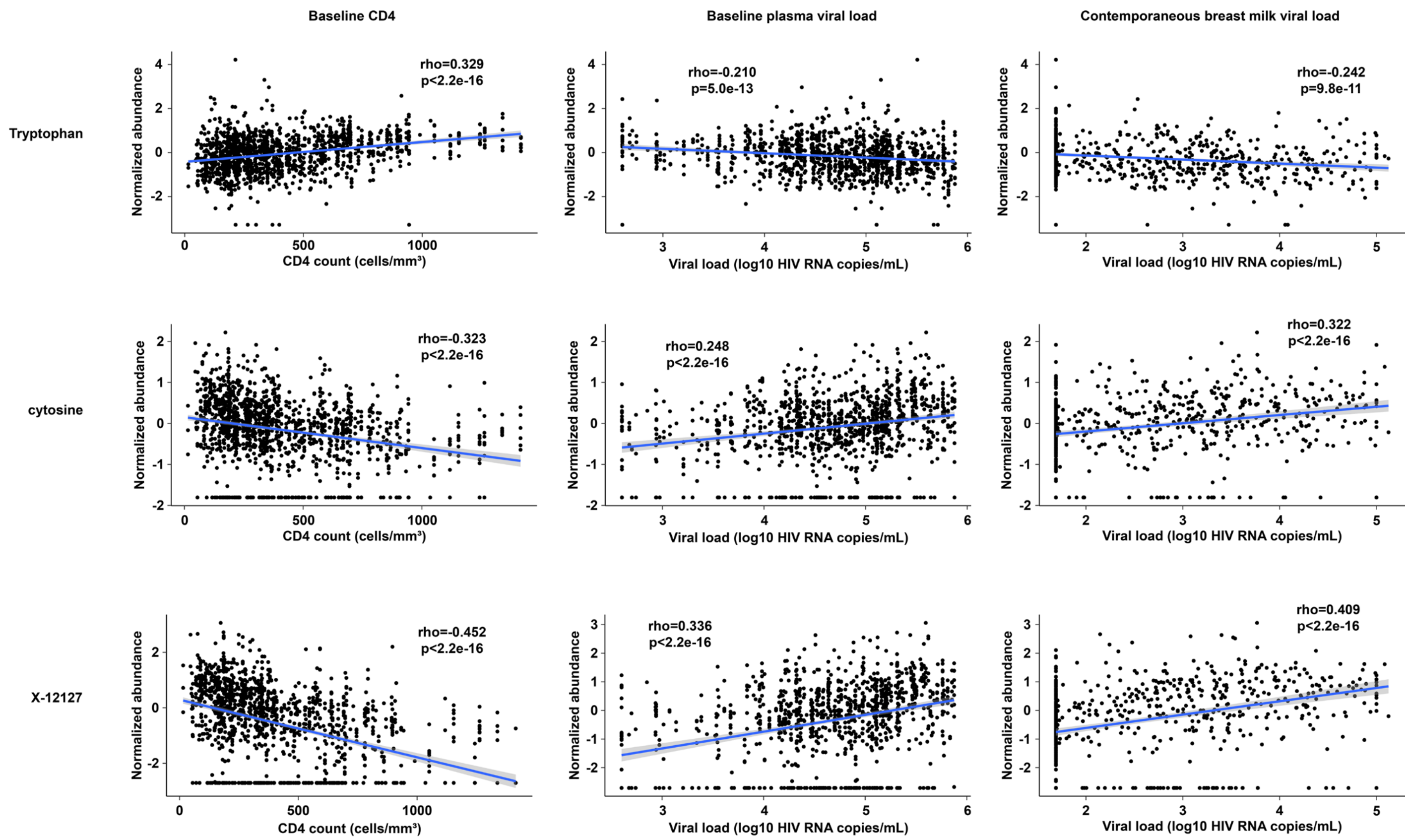


**Supplementary Figure 4**

Scatterplots of tryptophan, cytosine, and X-12127 versus baseline maternal CD4 cell count (cells/mm$^3$), baseline maternal viral load (log10 RNA copies/mL) or contemporaneous maternal breast milk viral load (log10 RNA copies/mL). Solid blue line indicates linear regression line and shaded gray area represents 95% confidence intervals. Spearman correlation coefficients and p-values are shown.

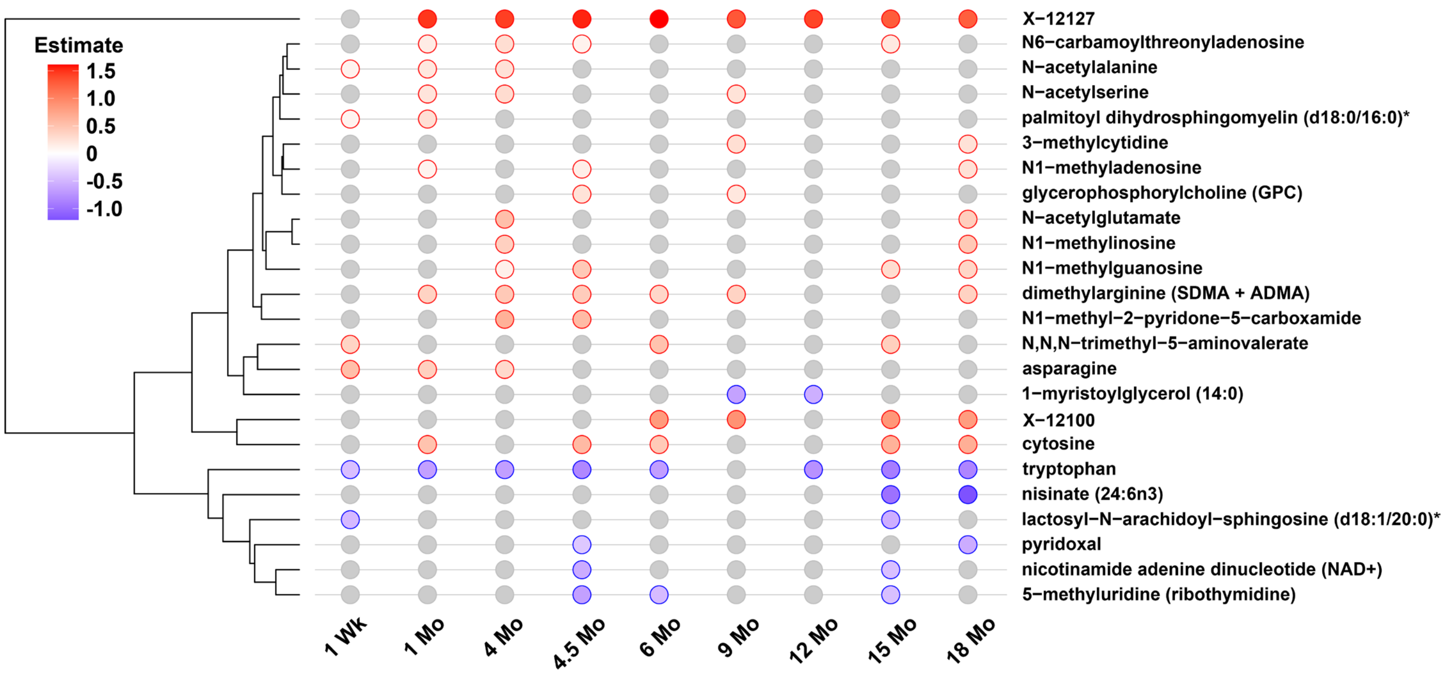


**Supplementary Figure 5**

Heatmap of mean importance values from random forests models of WLWH versus WWoH. Only features selected by RF models at more than one timepoint are shown. Coloring indicates the mixed model regression coefficient for each timepoint-feature combination.

a

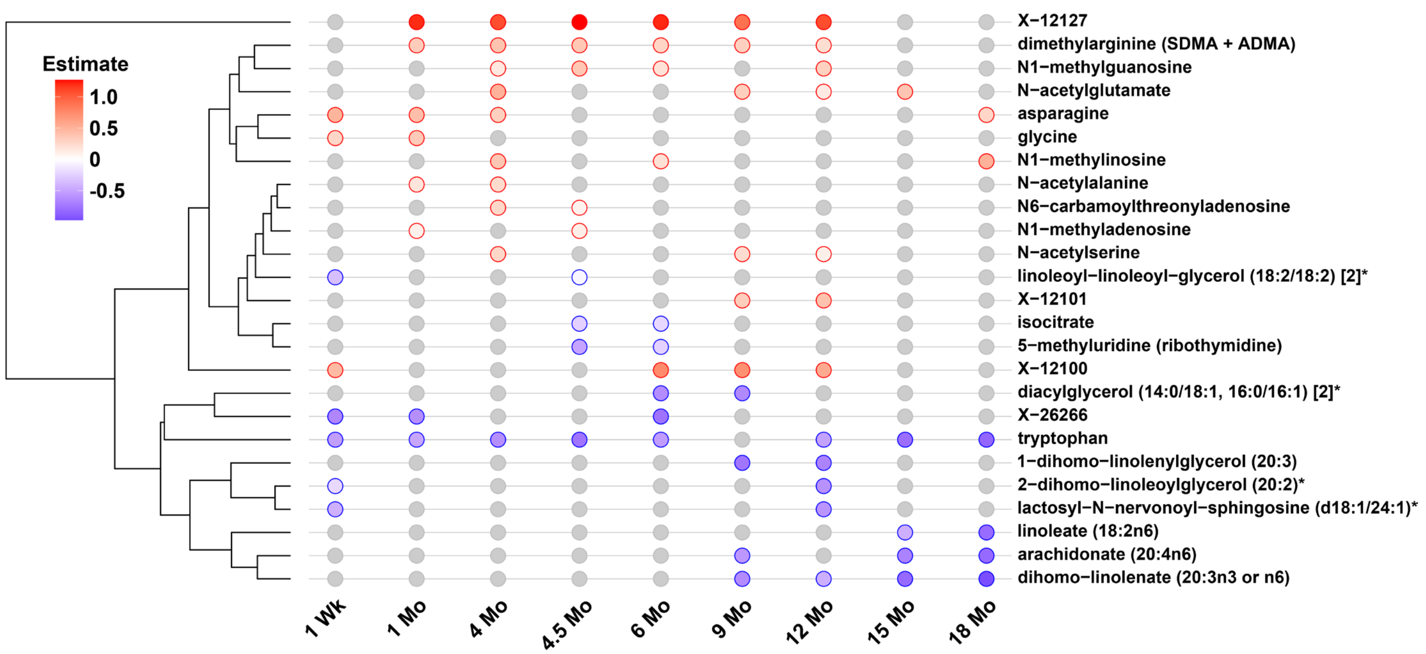


b

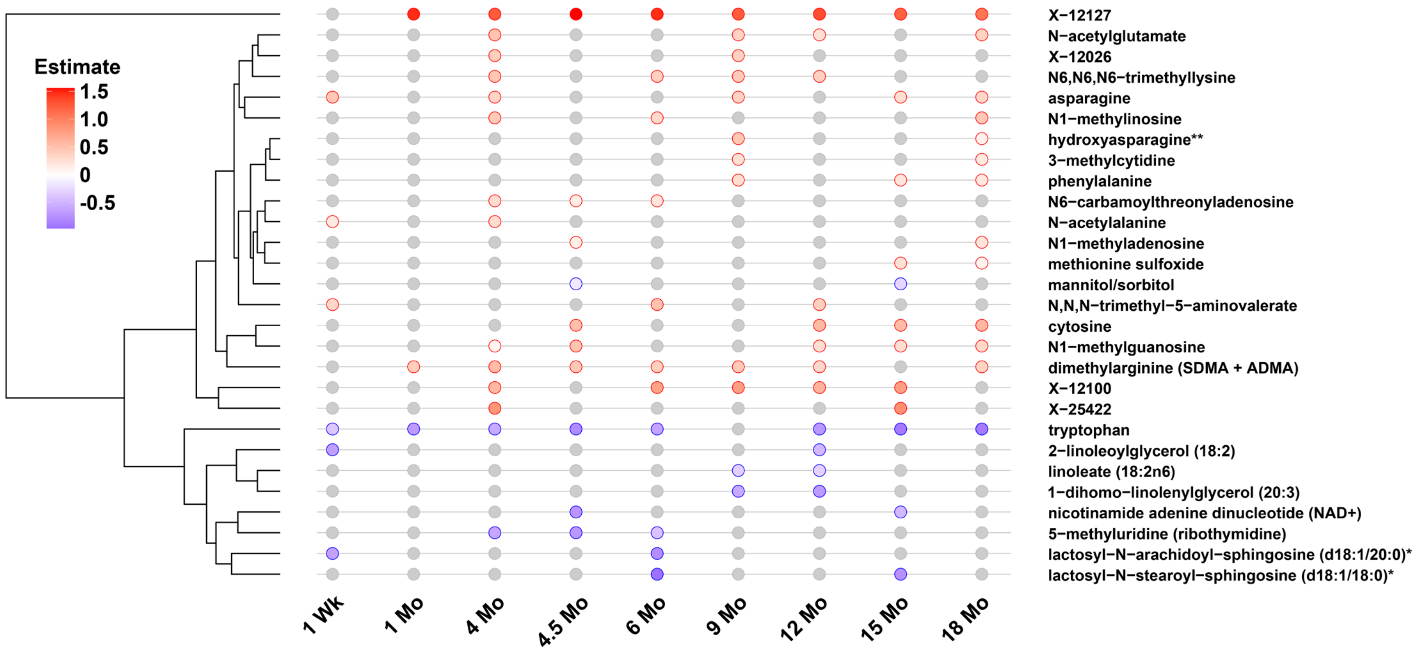


**Supplementary Figure 6**

Sub-analysis of WLWH versus WWoH among those whose children remained uninfected (a) or whose children survived the entire study course (b). Heatmap of mean importance values from random forests models of WLWH versus WWoH. Only features selected by RF models at more than one timepoint are shown. Coloring indicates the mixed model regression coefficient for each timepoint-feature combination.

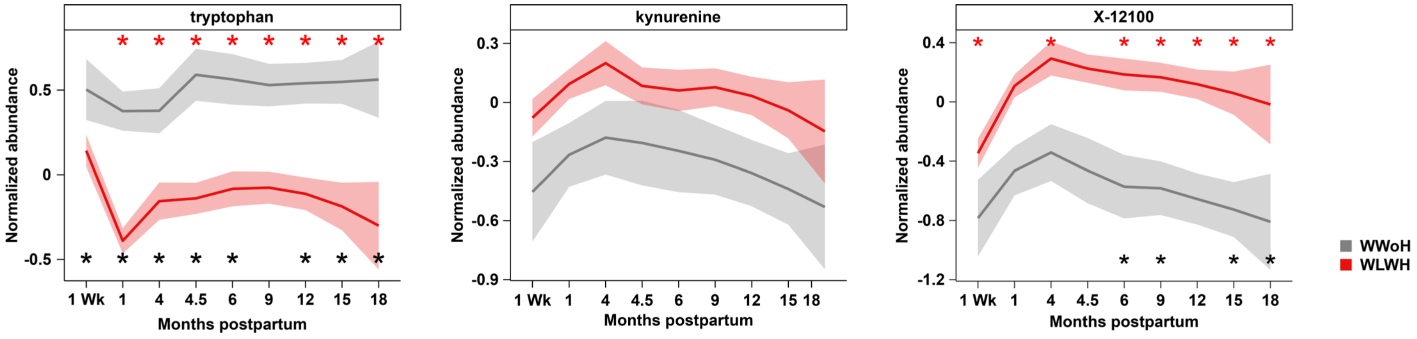


**Supplementary Figure 7**
Normalized abundances of tryptophan, kynurenine and X-12100 across the study course. Solid lines indicate mean abundances, and shaded areas denote 95% confidence intervals. Red asterisks along the top denote study visits at which the selected compound was differentially abundant in WLWH versus WWoH. Black asterisks along the bottom denote study visits at which the compound was selected as a predictive feature in the random forests modeling.

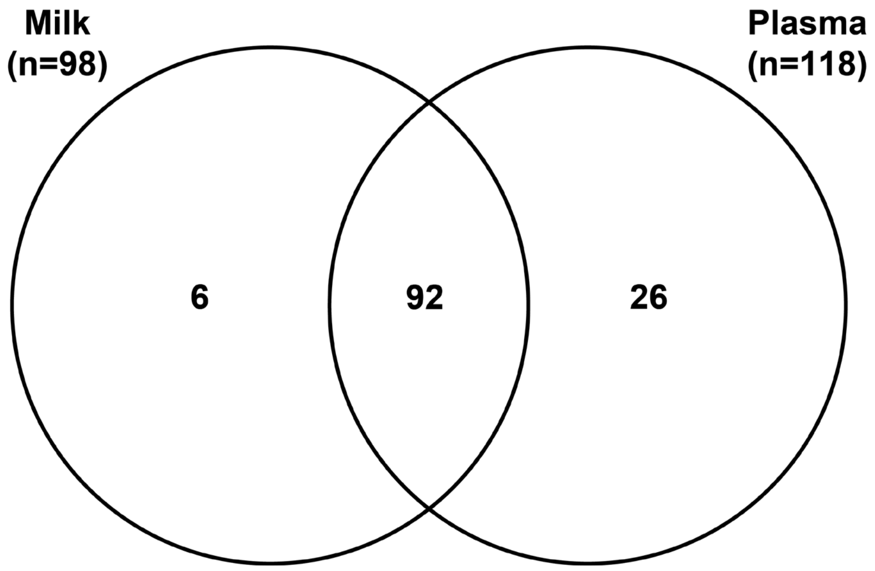


**Supplementary Figure 8**
Venn diagram showing the number of milk and plasma samples used in the quantitative KT analysis. The intersection shows the number of paired 4-month milk and plasma dyads that were used for the analysis of plasma to milk ratios.

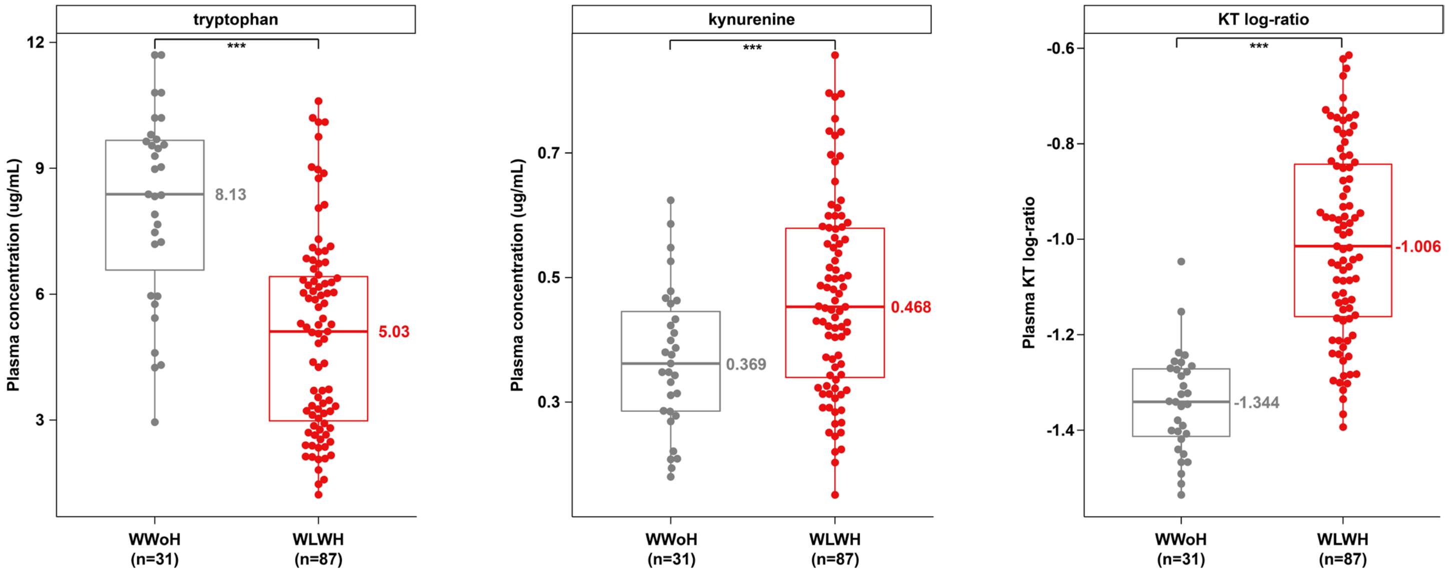


**Supplementary Figure 9**

Boxplots of plasma tryptophan, kynurenine, and KT log-ratio values in WLWH versus WWoH at the 4-month study visit, as calculated from the quantitative panel. * $p < 0.05$, ** $p < 0.01$, *** $p < 0.001$.

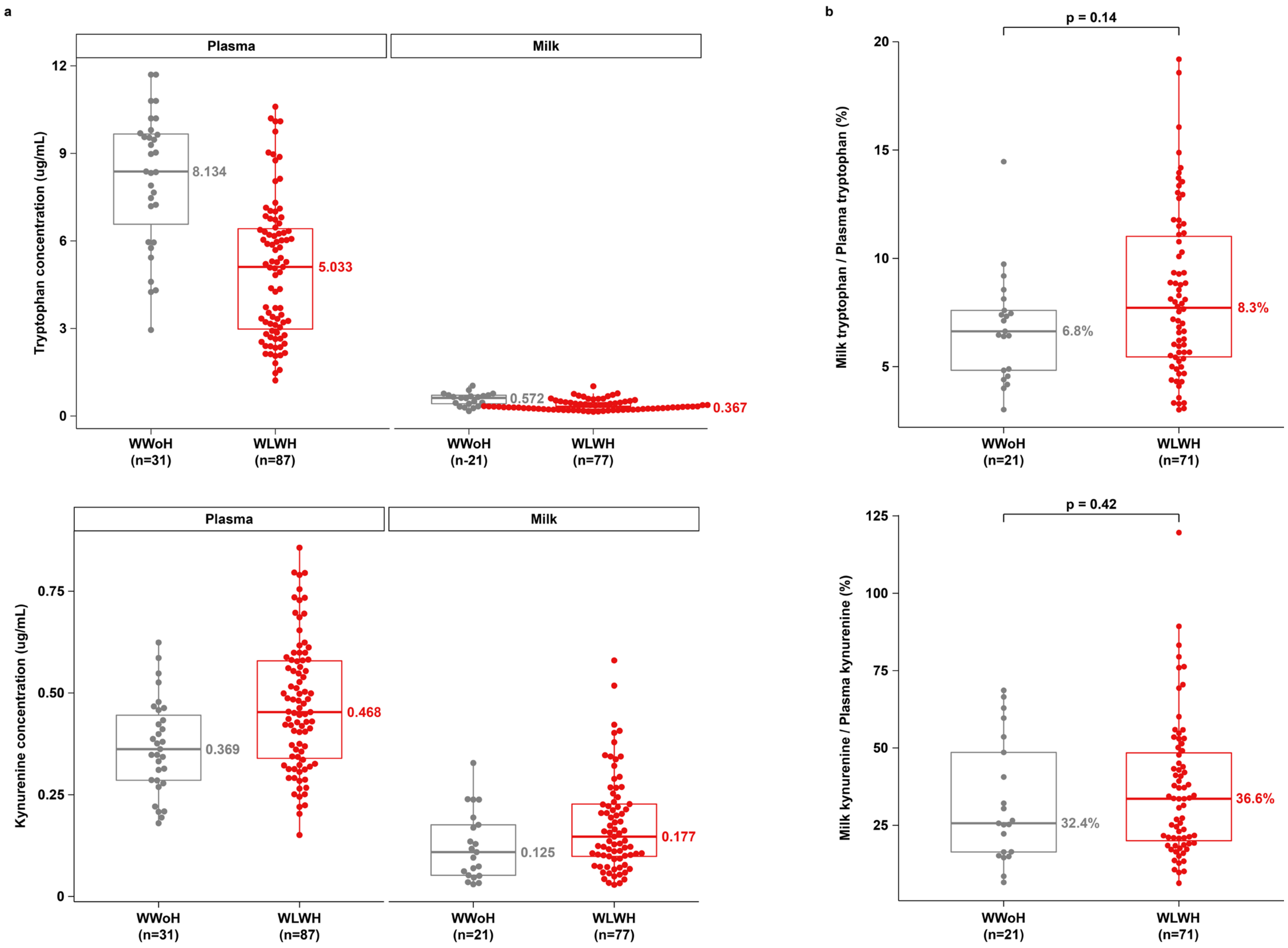


**Supplementary Figure 10**

Quantitative tryptophan and kynurenine levels in milk and plasma. a) Quantitative tryptophan (top) and kynurenine (bottom) levels by sample matrix and WLWH versus WWoH. B) Ratio between milk and plasma levels by WLWH versus WWoH for tryptophan (top) and kynurenine (bottom)

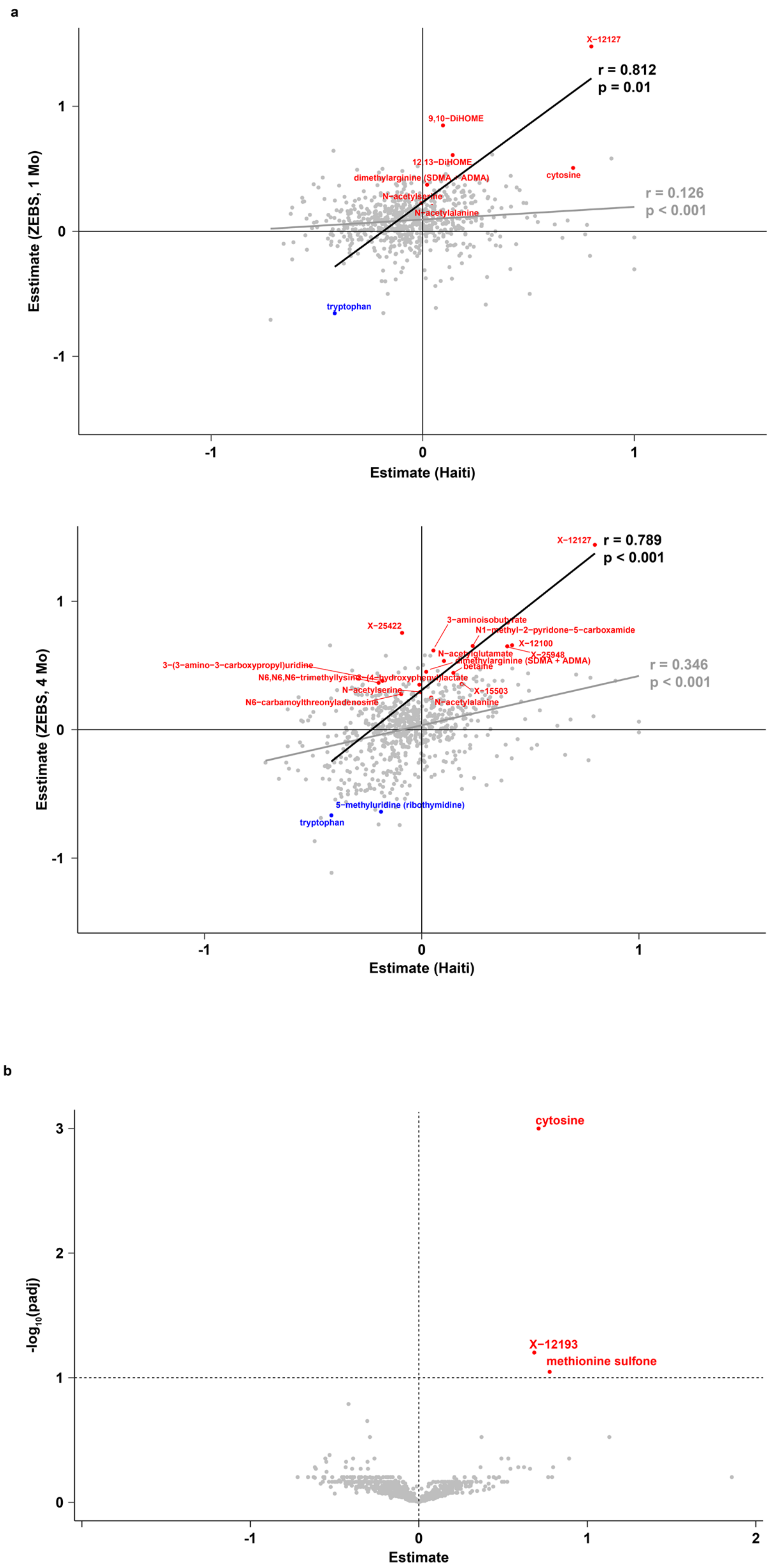


**Supplementary Figure 11**

Validation analyses in milk from an independent cohort of WLWH from Haiti. a) Correlation between linear regression estimates from the main cohort at the 1 Mo (upper panel) and 4 Mo (lower panel) timepoints and the Haiti validation cohort. Blue and red points denote metabolites that were significantly decreased and increased, respectively, in the main cohort analysis. The solid gray line shows a linear model fit for the regression estimates for all metabolites, and the solid black line shows a fit for only the significant metabolites. b) Volcano plot showing differences in metabolite abundances in the Haiti validation cohort. Blue and red points metabolites are significantly decreased and increased in WLWH versus WWoH, respectively.

**Supplementary Table 1**

Permutational multivariate analysis of variance (PERMANOVA) results.

| | Df | SumOfSqs | R2 | F | p | |
|---|---|---|---|---|---|---|
| Visit | 8 | 86468 | 0.03991 | 15.6126 | 0.001 | *** |
| HIV | 1 | 4170 | 0.00192 | 6.0237 | 0.001 | *** |
| Maternal CD4 count | 1 | 3021 | 0.00139 | 4.3634 | 0.001 | *** |
| Infant sex | 1 | 1420 | 0.00066 | 2.0504 | 0.01 | ** |
| Maternal age | 1 | 1660 | 0.00077 | 2.3971 | 0.008 | ** |
| Maternal education | 1 | 1899 | 0.00088 | 2.7429 | 0.002 | ** |
| Parity | 1 | 1951 | 0.0009 | 2.8176 | 0.003 | ** |
| Maternal BMI | 1 | 2515 | 0.00116 | 3.633 | 0.001 | *** |
| Residual | 1410 | 976137 | 0.45057 | | | |
| Total | 1425 | 1087205 | 0.50114 | | | |

**Supplementary Table 2**
Differences in global estimates of KT log-ratio by WLWH versus WWoH at each study visit.

| metabolite | contrast | Visit | estimate | SE | df | t.ratio | p.value | model | padj | dir |
|---|---|---|---|---|---|---|---|---|---|---|
| KTlogratio | WLWH - WWoH | 12 Mo | 0.505551 | 0.080696 | 1229.823 | 6.264918 | 5.15E-10 | LM | 4.63E-09 | up |
| KTlogratio | WLWH - WWoH | 1 Mo | 0.423063 | 0.070684 | 1042.889 | 5.985289 | 2.97E-09 | LM | 1.34E-08 | up |
| KTlogratio | WLWH - WWoH | 4.5 Mo | 0.486528 | 0.084997 | 1295.773 | 5.724046 | 1.29E-08 | LM | 3.87E-08 | up |
| KTlogratio | WLWH - WWoH | 4 Mo | 0.406286 | 0.074431 | 1120.366 | 5.458528 | 5.91E-08 | LM | 1.21E-07 | up |
| KTlogratio | WLWH - WWoH | 6 Mo | 0.422196 | 0.077699 | 1179.119 | 5.433742 | 6.7E-08 | LM | 1.21E-07 | up |
| KTlogratio | WLWH - WWoH | 9 Mo | 0.427452 | 0.080298 | 1223.287 | 5.323298 | 1.21E-07 | LM | 1.82E-07 | up |
| KTlogratio | WLWH - WWoH | 1 Wk | 0.363492 | 0.069955 | 1051.255 | 5.196077 | 2.44E-07 | LM | 3.14E-07 | up |
| KTlogratio | WLWH - WWoH | 15 Mo | 0.502062 | 0.097658 | 1386.547 | 5.141011 | 3.12E-07 | LM | 3.51E-07 | up |
| KTlogratio | WLWH - WWoH | 18 Mo | 0.536124 | 0.113774 | 1407.282 | 4.712167 | 2.69E-06 | LM | 2.69E-06 | up |

**Supplementary Table 3**

Demographics of women included in the quantitative tryptophan and kynurenine analyses. P-values were calculated using a Chi-square test for categorical variables and a two-sided Student's t-test for continuous variables.

| | WWoH | WLWH | p |
|---|---|---|---|
| **Quantitative milk KT** | | | |
| n | 21 | 77 | |
| Maternal Age (years), mean (SD) | 27.05 (7.40) | 26.17 (4.46) | 0.495 |
| Maternal BMI, mean (SD) | 23.23 (3.85) | 21.12 (3.27) | 0.013 |
| Parity, mean (SD) | 2.52 (1.94) | 2.05 (1.31) | 0.193 |
| Maternal education (years), mean (SD) | 7.95 (2.80) | 7.38 (3.15) | 0.45 |
| Food unavailable within the past month (n (%)) | 8 (38.1) | 20 (26.0) | 0.414 |
| Household electricity available (n (%)) | 13 (61.9) | 49 (63.6) | 1 |
| Log10 HIV RNA, mean(SD) | NaN (NA) | 4.72 (0.70) | NA |
| Maternal CD4 Count (cells/mm$^3$) | | | |
| Mean (SD) | 846.24 (261.60) | 330.00 (182.33) | <0.001 |
| <200 cells/mm$^3$ (n (%)) | 0 (0.0) | 19 (24.7) | |
| 200-349 cells/mm$^3$ (n (%)) | 1 (4.8) | 30 (39.0) | |
| >350 cells/mm$^3$ (n (%)) | 20 (95.2) | 28 (36.4) | <0.001 |
| Maternal CD8 Count (cells/mm$^3$), mean (SD) | 623.86 (270.03) | 782.11 (420.31) | 0.106 |
| Maternal CD3 Count (cells/mm$^3$), mean (SD) | 1568.48 (515.18) | 1169.87 (512.35) | 0.002 |
| Caesarean Section (%) | 1 (4.8) | 2 (2.6) | 1 |
| Nevirapine at Delivery (%) | NA | 21 (100) | NA |
| Gestational Age at Delivery (weeks), mean (SD) | 39.59 (6.06) | 37.90 (4.12) | 0.14 |
| Infant Birth Weight (kg), mean (SD) | 3.12 (0.55) | 2.97 (0.55) | 0.264 |
| Infant Male Sex = n (%) | 6 (28.6) | 37 (48.1) | 0.178 |
| 12-month CD4 Count (cells/mm$^3$), mean (SD) | 947.47 (319.49) | 345.65 (222.46) | <0.001 |
| Child death (%) | 0 (0.0) | 41 (53.2)* | NA |
| Maternal death (%) | 0 (0.0) | 9 (11.7) | 0.223 |
| | | | |
| | **WWoH** | **WLWH** | p |
| **Quantitative plasma KT** | | | |
| n | 31 | 87 | |
| Maternal Age (years), mean (SD) | 25.97 (6.68) | 25.66 (4.46) | 0.771 |
| Maternal BMI, mean (SD) | 22.22 (3.77) | 21.31 (3.21) | 0.199 |
| Parity, mean (SD) | 2.55 (1.96) | 2.45 (1.67) | 0.785 |
| Maternal education (years), mean (SD) | 7.39 (2.80) | 7.39 (3.21) | 0.995 |
| Food unavailable within the past month (n (%)) | 10 (32.3) | 19 (21.8) | 0.361 |
| Household electricity available (n (%)) | 21 (67.7) | 53 (60.9) | 0.647 |
| Log10 HIV RNA, mean(SD) | NaN (NA) | 4.62 (0.72) | NA |
| Maternal CD4 Count (cells/mm$^3$) | | | |
| Mean (SD) | 840.87 (264.76) | 354.28 (200.19) | <0.001 |
| <200 cells/mm$^3$ (n (%)) | 0 (0.0) | 22 (25.3) | |
| 200-349 cells/mm$^3$ (n (%)) | 2 (6.5) | 27 (31.0) | |
| >350 cells/mm$^3$ (n (%)) | 29 (93.5) | 38 (43.7) | |
| Maternal CD8 Count (cells/mm$^3$), mean (SD) | 569.87 (247.40) | 793.78 (415.36) | 0.006 |
| Maternal CD3 Count (cells/mm$^3$), mean (SD) | 1491.87 (475.48) | 1206.07 (530.32) | 0.009 |

| | | | |
|---|---|---|---|
| Caesarean Section (%) | 1 (3.2) | 2 (2.3) | 1 |
| Nevirapine at Delivery (%) | NA | 30 (96.8) | NA |
| Gestational Age at Delivery (weeks), mean (SD) | 38.86 (5.86) | 37.81 (4.01) | 0.278 |
| Infant Birth Weight (kg), mean (SD) | 3.31 (1.34) | 3.00 (0.53) | 0.068 |
| Infant Male Sex = n (%) | 11 (35.5) | 44 (50.6) | 0.216 |
| 12-month CD4 Count (cells/mm$^3$), mean (SD) | 943.59 (358.25) | 369.73 (228.46) | <0.001 |
| Child death (%) | 1 (3.2) | 39 (44.8)* | NA |
| Maternal death (%) | 0 (0.0) | 9 (10.3) | 0.142 |
| | | | |
| | **WWoH** | **WLWH** | p |
| **Quantitative plasma + milk KT** | | | |
| n | 21 | 71 | |
| Maternal Age (years), mean (SD) | 27.05 (7.40) | 26.15 (4.41) | 0.493 |
| Maternal BMI, mean (SD) | 23.23 (3.85) | 21.24 (3.26) | 0.021 |
| Parity, mean (SD) | 2.52 (1.94) | 2.07 (1.30) | 0.217 |
| Maternal education (years), mean (SD) | 7.95 (2.80) | 7.42 (3.16) | 0.491 |
| Food unavailable within the past month (n (%)) | 8 (38.1) | 17 (23.9) | 0.317 |
| Household electricity available (n (%)) | 13 (61.9) | 44 (62.0) | 1 |
| Log10 HIV RNA, mean(SD) | NaN (NA) | 4.71 (0.71) | NA |
| Maternal CD4 Count (cells/mm$^3$) | | | |
| Mean (SD) | 846.24 (261.60) | 337.55 (187.30) | <0.001 |
| <200 cells/mm$^3$ (n (%)) | 0 (0.0) | 18 (25.4) | |
| 200-349 cells/mm$^3$ (n (%)) | 1 (4.8) | 25 (35.2) | |
| >350 cells/mm$^3$ (n (%)) | 20 (95.2) | 28 (39.4) | |
| Maternal CD8 Count (cells/mm$^3$), mean (SD) | 623.86 (270.03) | 801.99 (431.65) | 0.078 |
| Maternal CD3 Count (cells/mm$^3$), mean (SD) | 1568.48 (515.18) | 1197.20 (524.63) | 0.005 |
| Caesarean Section (%) | 1 (4.8) | 2 (2.8) | 1 |
| Nevirapine at Delivery (%) | NA | 21 (100) | NA |
| Gestational Age at Delivery (weeks), mean (SD) | 39.59 (6.06) | 37.64 (4.15) | 0.096 |
| Infant Birth Weight (kg), mean (SD) | 3.12 (0.55) | 2.98 (0.57) | 0.302 |
| Infant Male Sex = n (%) | 6 (28.6) | 35 (49.3) | 0.153 |
| 12-month CD4 Count (cells/mm$^3$), mean (SD) | 947.47 (319.49) | 355.22 (229.21) | <0.001 |
| Child death (%) | 0 (0.0) | 35 (49.3)* | NA |
| Maternal death (%) | 0 (0.0) | 8 (11.3) | 0.242 |

**Supplementary Table 4**

Summary statistics of quantitative tryptophan and kynurenine levels in WLWH and WWoH from milk and plasma samples taken at the 4-month timepoint.

| | **WLWH** | **WWoH** | **p** |
|---|---|---|---|
| **Tryptophan, mean (sd)** | | | |
| Milk | 0.367 (0.177) | 0.572 (0.222) | 0.00082 |
| Plasma | 5.033 (2.357) | 8.134 (2.298) | 1.29E-07 |
| Milk / Plasma Ratio | 0.083 (0.038) | 0.068 (0.025) | 0.14 |
| **Kynurenine, mean (sd)** | | | |
| Milk | 0.177 (0.117) | 0.125 (0.084) | 0.028 |
| Plasma | 0.468 (0.158) | 0.369 (0.115) | 0.00065 |
| Milk / Plasma Ratio | 0.366 (0.220) | 0.324 (0.199) | 0.42 |
| **KT log-ratio, mean (sd)** | | | |
| Milk | -0.358 (0.254) | -0.726 (0.308) | 5.78E-05 |
| Plasma | -1.006 (0.199) | -1.344 (0.107) | 4.99E-19 |
