# Supplementary material for: Altered milk tryptophan and tryptophan metabolites in women living with HIV
Source: Nat Commun. 2025 Oct 28;16:9437. doi: 10.1038/s41467-025-64566-w (PMC12568960; doi:10.1038/s41467-025-64566-w)
Supplement: Supplementary file 3 — Description of Additional Supplementary Files [file 41467_2025_64566_MOESM3_ESM.pdf]

Description of Additional Supplementary Files:

Supplementary Data 1
Estimated effects of WLWH versus WWoH from linear regression models, averaged across study visits. Results are reported as t-ratios, which are the estimates of the regression coefficients divided by their standard error.

Supplementary Data 2
Estimated effects of WLWH versus WWoH from linear regression models, stratified by study visit. Results are reported as t-ratios, which are the estimates of the regression coefficients divided by their standard error.

Supplementary Data 3
Spearman correlation between metabolites and maternal baseline CD4 and plasma viral load and contemporaneous breast milk viral load.

Supplementary Data 4
Normalized abundances of all significant metabolites either by linear regression or random forests across the study course. Solid lines indicate mean abundances, and shaded areas denote 95% confidence intervals. Red asterisks along the top denote study visits at which the selected compound was differentially abundant in WLWH versus WWoH. Black asterisks along the bottom denote study visits at which the compound was selected as a predictive feature in the random forests modeling.

Supplementary Data 5
Mean feature importance values from random forests modeling of WLWH versus WWoH for each study visit. Only the features selected and included in the final sparse models are shown.

Supplementary Data 6
Estimated effects of WLWH whose children remained uninfected versus WWoH from linear regression models, averaged across study visits. Results are reported as t-ratios, which are the estimates of the regression coefficients divided by their standard error.

Supplementary Data 7
Estimated effects of WLWH whose children remained uninfected versus WWoH from linear regression models, stratified by study visit. Results are reported as t-ratios, which are the estimates of the regression coefficients divided by their standard error.

Supplementary Data 8
Estimated effects of WLWH whose children remained uninfected versus WWoH from linear regression models, averaged across study visits. Results are reported as t-ratios, which are the estimates of the regression coefficients divided by their standard error.

Supplementary Data 9
Estimated effects of WLWH whose children remained uninfected versus WWoH from linear regression models, stratified by study visit. Results are reported as t-ratios, which are the estimates of the regression coefficients divided by their standard error.

Supplementary Data 10

All quantitative tryptophan and kynurenine levels in WLWH and WWoH from milk and plasma samples taken at the 4-month timepoint.

Supplementary Data 11

Estimated effects of WLWH versus WWoH from linear regression models in the Haiti validation cohort. Results are reported as t-ratios, which are the estimates of the regression coefficients divided by their standard error.
