# Supplementary material for: Altered milk tryptophan and tryptophan metabolites in women living with HIV
Source: Nat Commun. 2025 Oct 28;16:9437. doi: 10.1038/s41467-025-64566-w (PMC12568960; doi:10.1038/s41467-025-64566-w)
Supplement: Supplementary file 7 — Supplementary Data 4 [file 41467_2025_64566_MOESM7_ESM.pdf]

Amino Acid

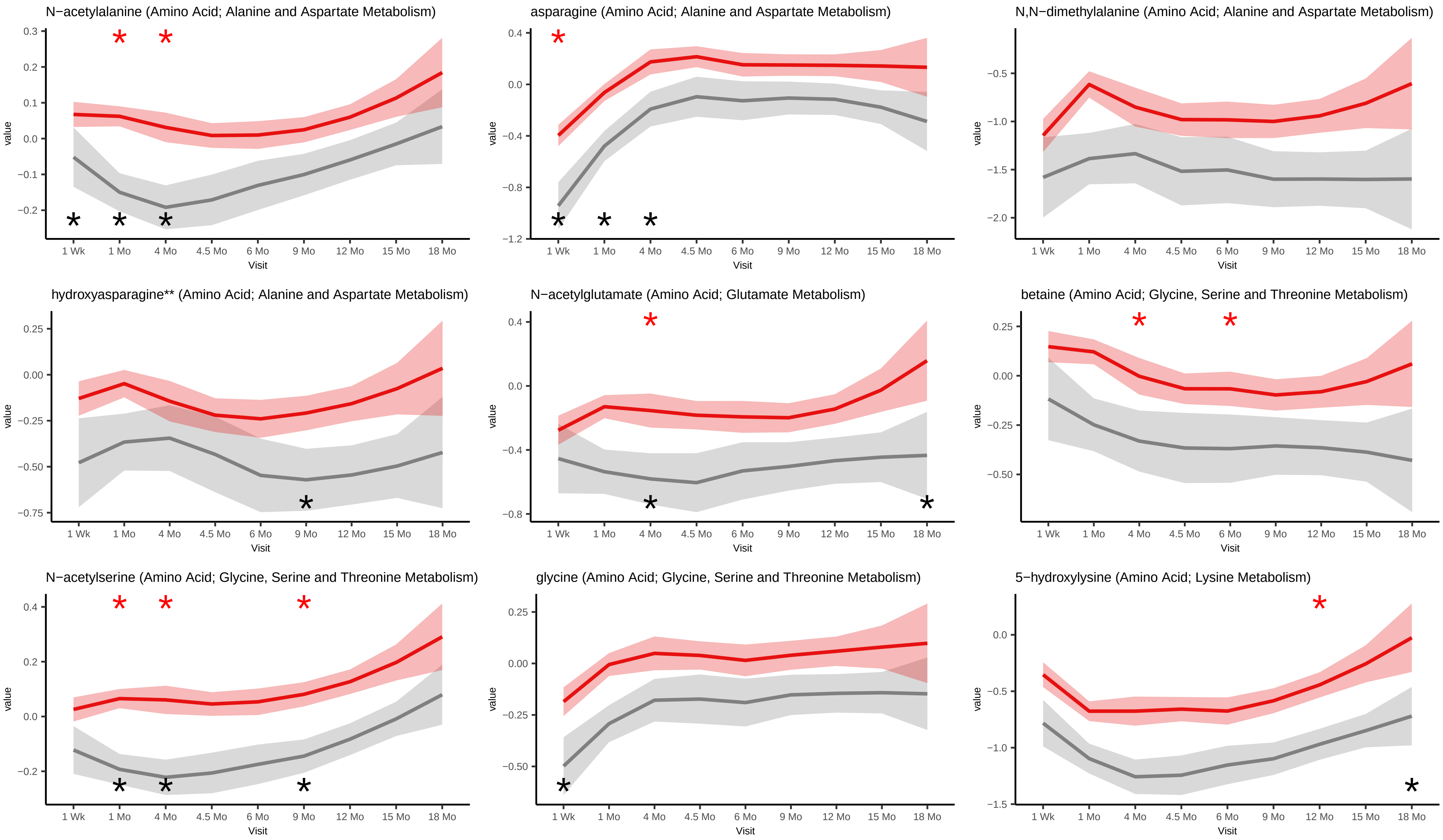
N−acetylalanine (Amino Acid; Alanine and Aspartate Metabolism)
asparagine (Amino Acid; Alanine and Aspartate Metabolism)
N,N−dimethylalanine (Amino Acid; Alanine and Aspartate Metabolism)
hydroxyasparagine** (Amino Acid; Alanine and Aspartate Metabolism)
N−acetylglutamate (Amino Acid; Glutamate Metabolism)
betaine (Amino Acid; Glycine, Serine and Threonine Metabolism)
N−acetylserine (Amino Acid; Glycine, Serine and Threonine Metabolism)
glycine (Amino Acid; Glycine, Serine and Threonine Metabolism)
5−hydroxylysine (Amino Acid; Lysine Metabolism)
value
Visit
1 Wk
1 Mo
4 Mo
4.5 Mo
6 Mo
9 Mo
12 Mo
15 Mo
18 Mo

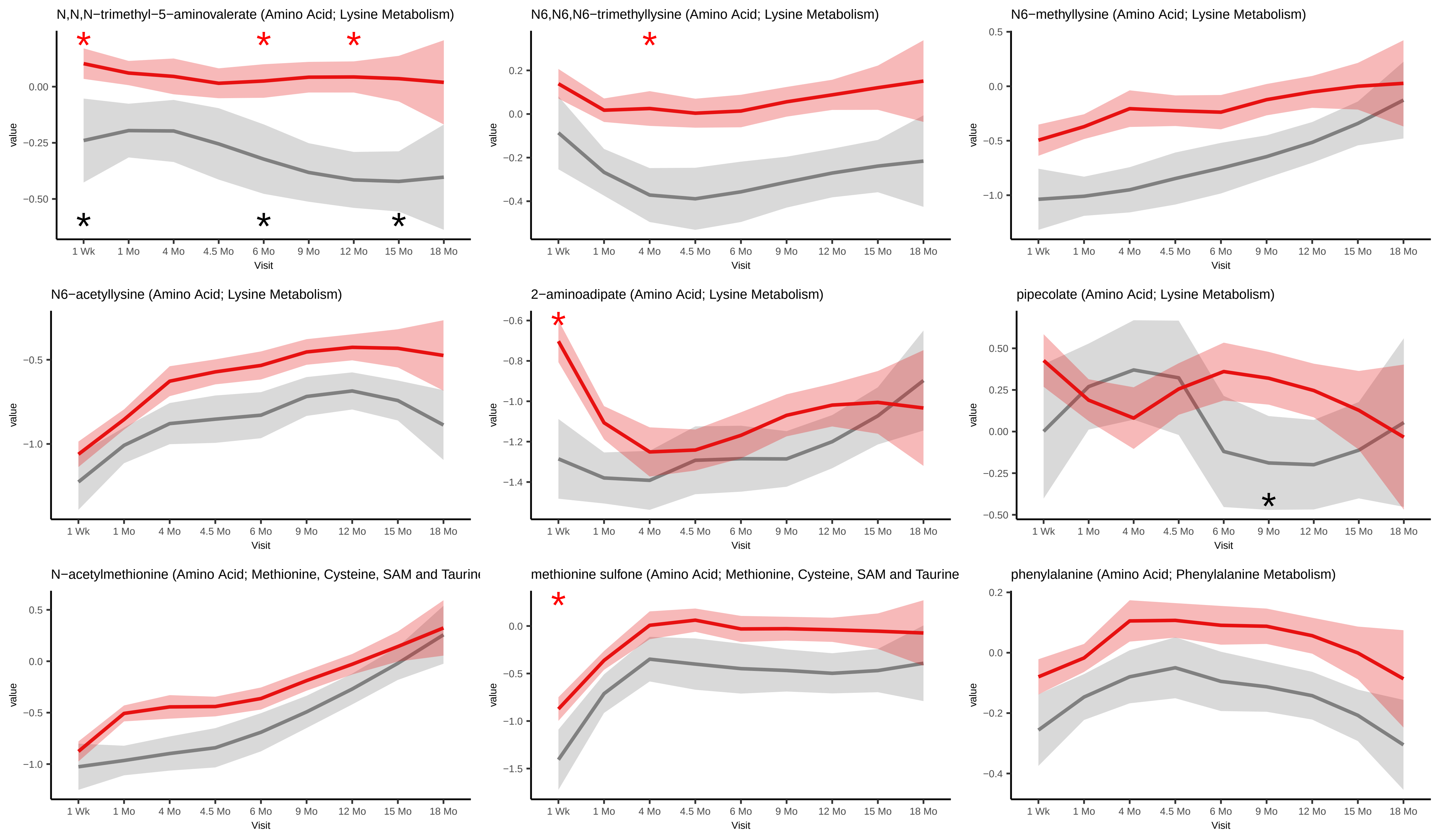

N,N,N−trimethyl−5−aminovalerate (Amino Acid; Lysine Metabolism)
0.00
−0.25
−0.50
value
1 Wk
1 Mo
4 Mo
4.5 Mo
6 Mo
9 Mo
12 Mo
15 Mo
18 Mo
Visit
N6,N6,N6−trimethyllysine (Amino Acid; Lysine Metabolism)
0.2
0.0
−0.2
−0.4
value
1 Wk
1 Mo
4 Mo
4.5 Mo
6 Mo
9 Mo
12 Mo
15 Mo
18 Mo
Visit
N6−methyllysine (Amino Acid; Lysine Metabolism)
0.5
0.0
−0.5
−1.0
value
1 Wk
1 Mo
4 Mo
4.5 Mo
6 Mo
9 Mo
12 Mo
15 Mo
18 Mo
Visit
N6−acetyllysine (Amino Acid; Lysine Metabolism)
−0.5
−1.0
value
1 Wk
1 Mo
4 Mo
4.5 Mo
6 Mo
9 Mo
12 Mo
15 Mo
18 Mo
Visit
2−aminoadipate (Amino Acid; Lysine Metabolism)
−0.6
−0.8
−1.0
−1.2
−1.4
value
1 Wk
1 Mo
4 Mo
4.5 Mo
6 Mo
9 Mo
12 Mo
15 Mo
18 Mo
Visit
pipecolate (Amino Acid; Lysine Metabolism)
0.50
0.25
0.00
−0.25
−0.50
value
1 Wk
1 Mo
4 Mo
4.5 Mo
6 Mo
9 Mo
12 Mo
15 Mo
18 Mo
Visit
N−acetylmethionine (Amino Acid; Methionine, Cysteine, SAM and Taurin
0.5
0.0
−0.5
−1.0
value
1 Wk
1 Mo
4 Mo
4.5 Mo
6 Mo
9 Mo
12 Mo
15 Mo
18 Mo
Visit
methionine sulfone (Amino Acid; Methionine, Cysteine, SAM and Taurine
0.0
−0.5
−1.0
−1.5
value
1 Wk
1 Mo
4 Mo
4.5 Mo
6 Mo
9 Mo
12 Mo
15 Mo
18 Mo
Visit
phenylalanine (Amino Acid; Phenylalanine Metabolism)
0.2
0.0
−0.2
−0.4
value
1 Wk
1 Mo
4 Mo
4.5 Mo
6 Mo
9 Mo
12 Mo
15 Mo
18 Mo
Visit

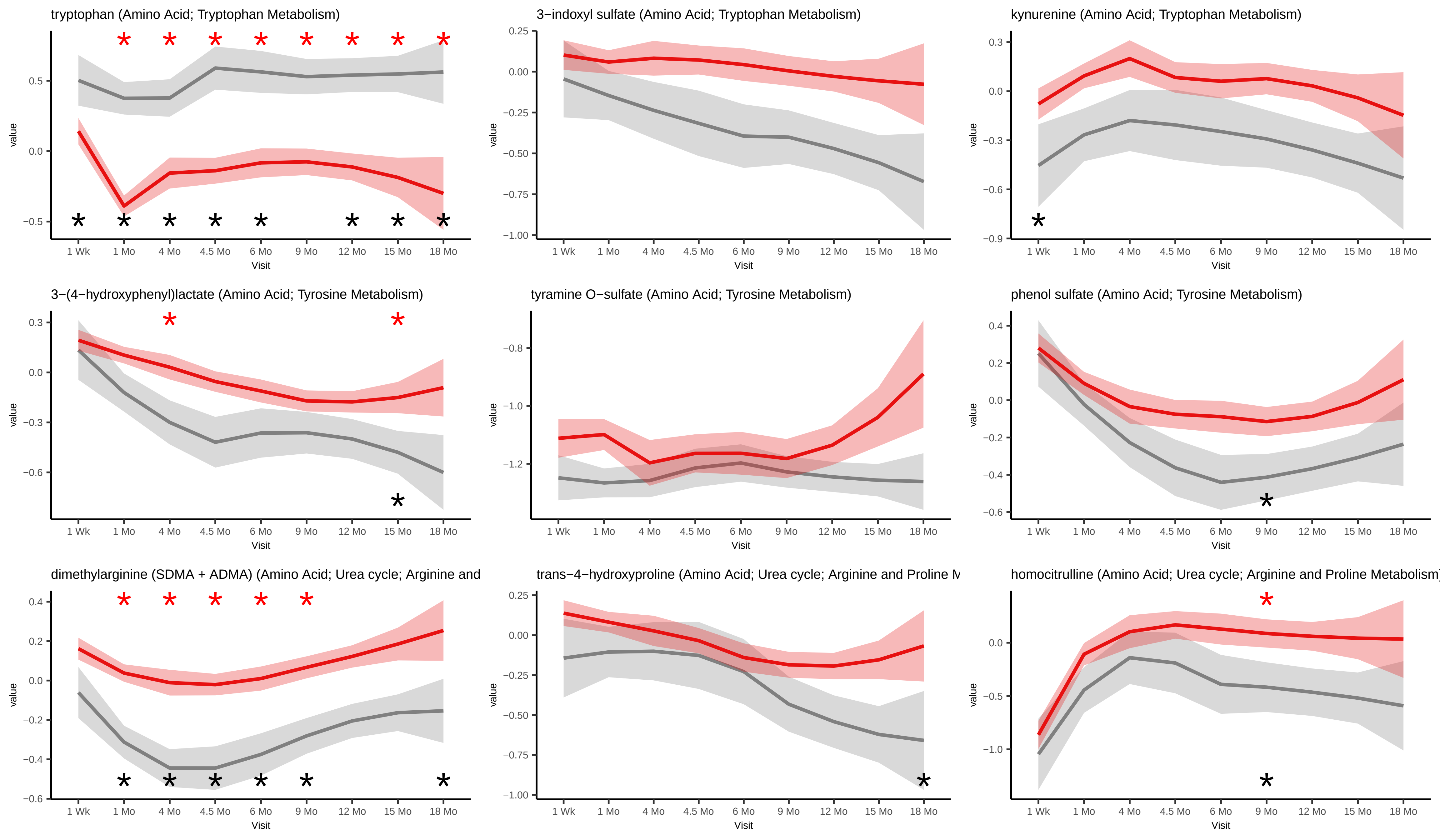

tryptophan (Amino Acid; Tryptophan Metabolism)
3−indoxyl sulfate (Amino Acid; Tryptophan Metabolism)
kynurenine (Amino Acid; Tryptophan Metabolism)
3−(4−hydroxyphenyl)lactate (Amino Acid; Tyrosine Metabolism)
tyramine O−sulfate (Amino Acid; Tyrosine Metabolism)
phenol sulfate (Amino Acid; Tyrosine Metabolism)
dimethylarginine (SDMA + ADMA) (Amino Acid; Urea cycle; Arginine and
trans−4−hydroxyproline (Amino Acid; Urea cycle; Arginine and Proline M
homocitrulline (Amino Acid; Urea cycle; Arginine and Proline Metabolism)
value
Visit
1 Wk
1 Mo
4 Mo
4.5 Mo
6 Mo
9 Mo
12 Mo
15 Mo
18 Mo

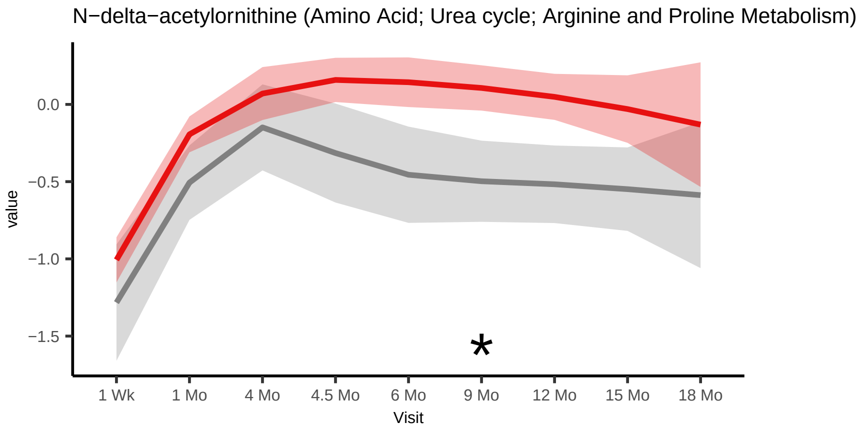
N−delta−acetylornithine (Amino Acid; Urea cycle; Arginine and Proline Metabolism)
value
0.0
−0.5
−1.0
−1.5
*
1 Wk
1 Mo
4 Mo
4.5 Mo
6 Mo
9 Mo
12 Mo
15 Mo
18 Mo
Visit

Carbohydrate

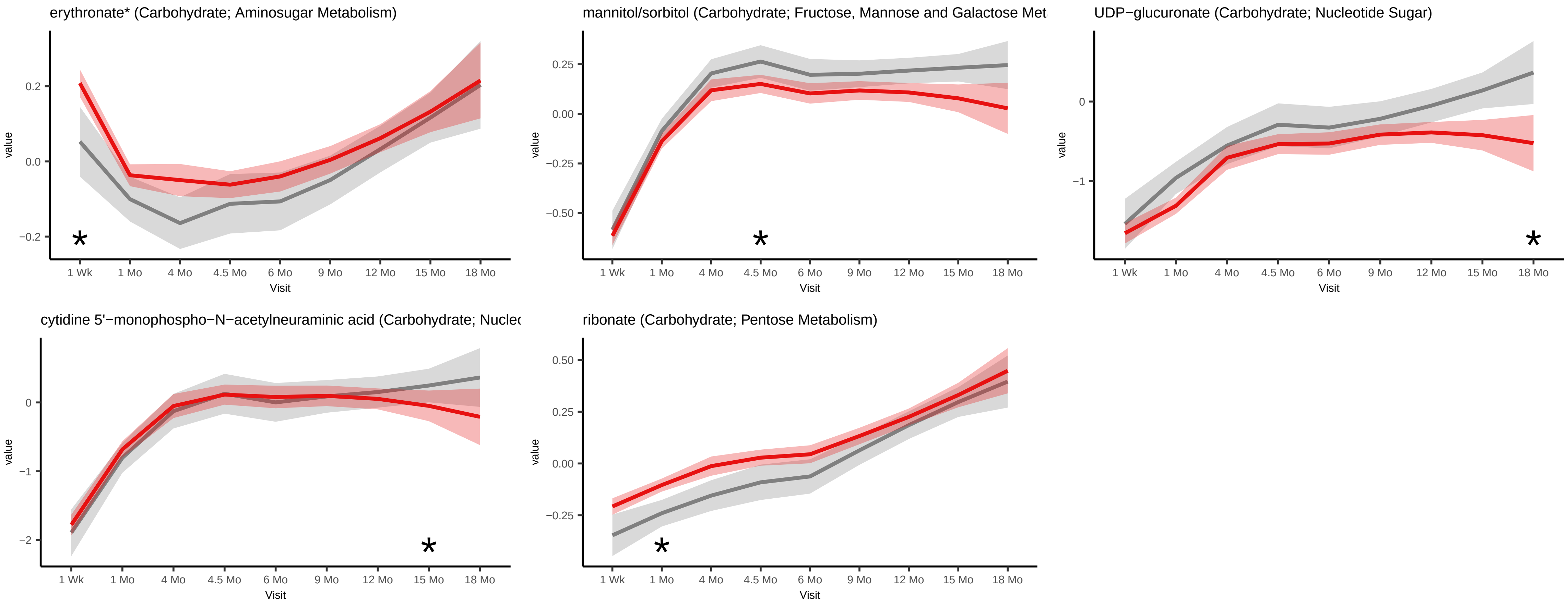
erythronate* (Carbohydrate; Aminosugar Metabolism)
mannitol/sorbitol (Carbohydrate; Fructose, Mannose and Galactose Met
UDP−glucuronate (Carbohydrate; Nucleotide Sugar)
cytidine 5'−monophospho−N−acetylneuraminic acid (Carbohydrate; Nucleo
ribonate (Carbohydrate; Pentose Metabolism)
value
Visit
1 Wk
1 Mo
4 Mo
4.5 Mo
6 Mo
9 Mo
12 Mo
15 Mo
18 Mo
*

Cofactors and Vitamins

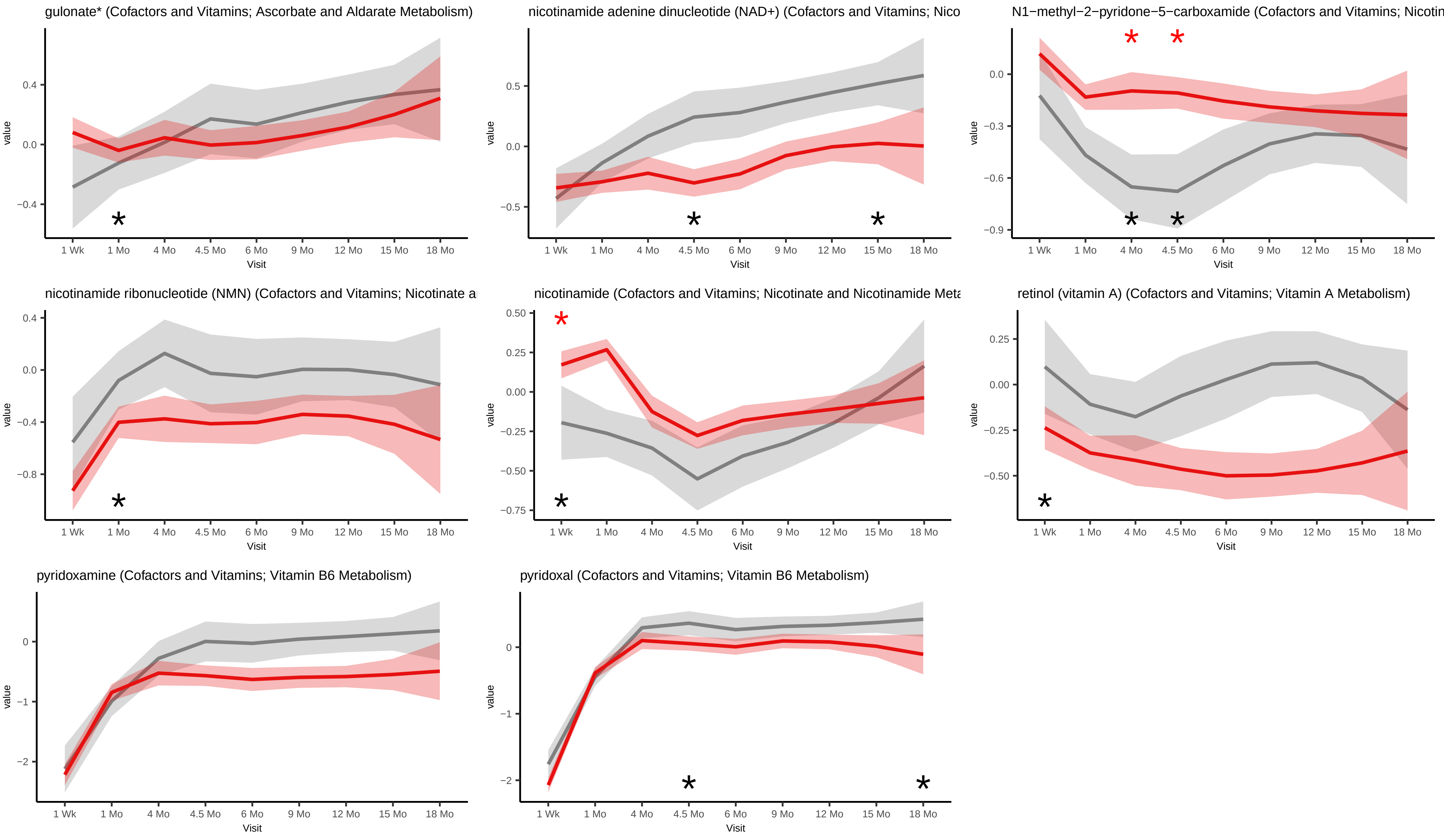
gulonate* (Cofactors and Vitamins; Ascorbate and Aldarate Metabolism)
nicotinamide adenine dinucleotide (NAD+) (Cofactors and Vitamins; Nico
N1−methyl−2−pyridone−5−carboxamide (Cofactors and Vitamins; Nicotin
nicotinamide ribonucleotide (NMN) (Cofactors and Vitamins; Nicotinate a
nicotinamide (Cofactors and Vitamins; Nicotinate and Nicotinamide Meta
retinol (vitamin A) (Cofactors and Vitamins; Vitamin A Metabolism)
pyridoxamine (Cofactors and Vitamins; Vitamin B6 Metabolism)
pyridoxal (Cofactors and Vitamins; Vitamin B6 Metabolism)
value
Visit
1 Wk
1 Mo
4 Mo
4.5 Mo
6 Mo
9 Mo
12 Mo
15 Mo
18 Mo
*

Energy

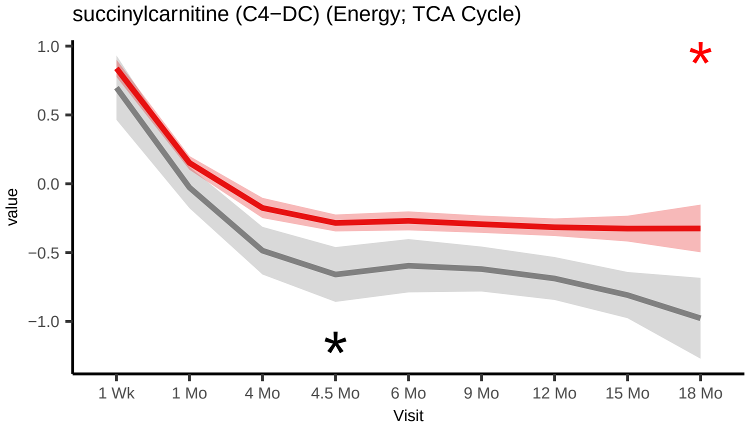

succinylcarnitine (C4−DC) (Energy; TCA Cycle)
value
1.0
0.5
0.0
−0.5
−1.0
*
*
1 Wk
1 Mo
4 Mo
4.5 Mo
6 Mo
9 Mo
12 Mo
15 Mo
18 Mo
Visit

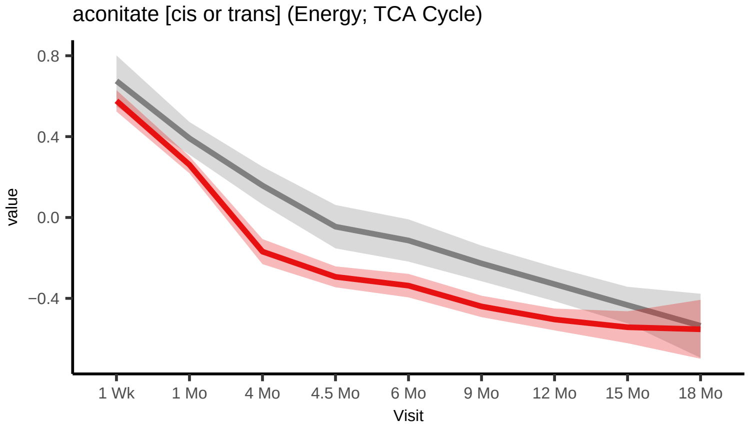

aconitate [cis or trans] (Energy; TCA Cycle)
value
0.8
0.4
0.0
−0.4
1 Wk
1 Mo
4 Mo
4.5 Mo
6 Mo
9 Mo
12 Mo
15 Mo
18 Mo
Visit

Lipid

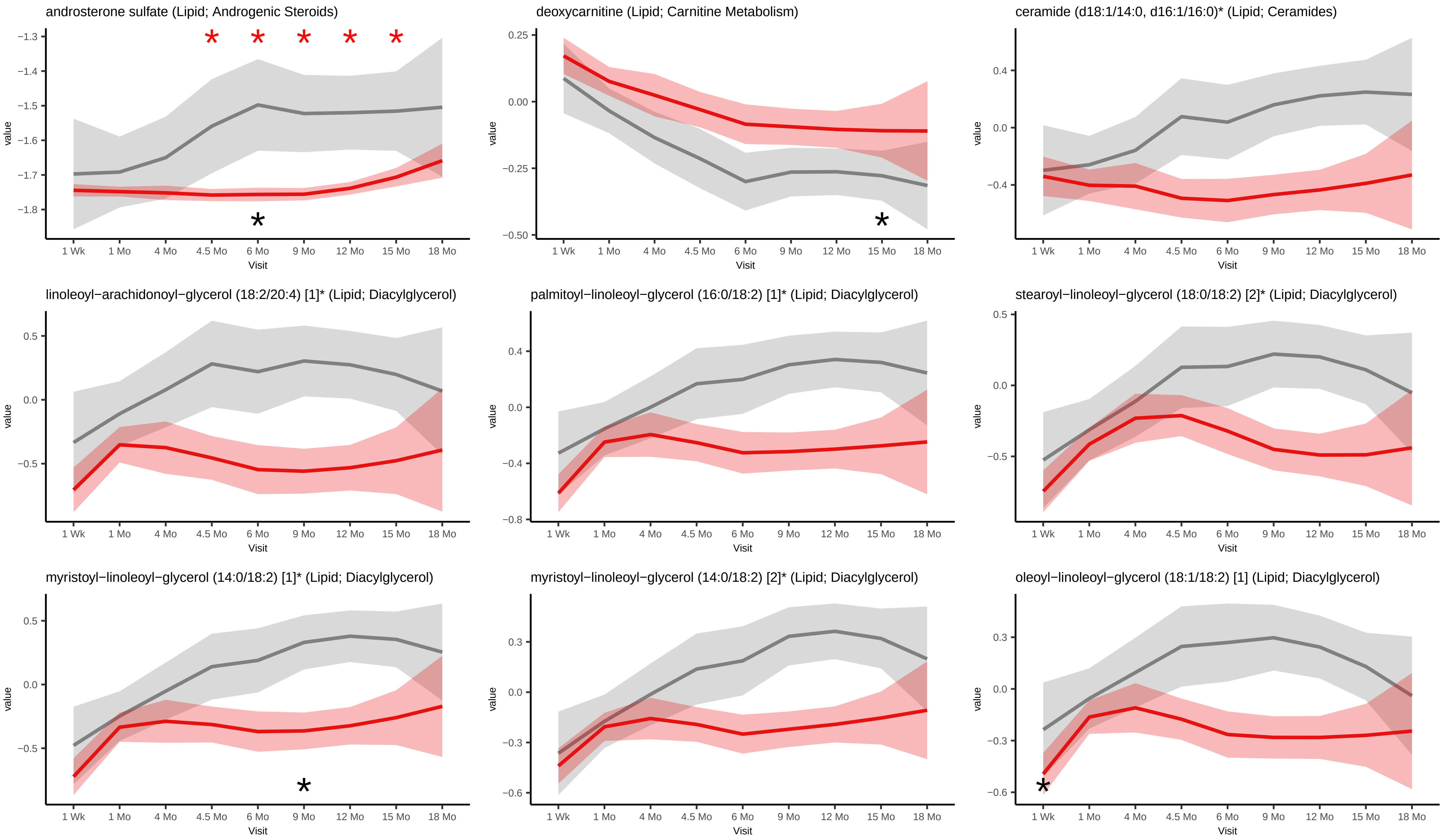

androsterone sulfate (Lipid; Androgenic Steroids)
deoxycarnitine (Lipid; Carnitine Metabolism)
ceramide (d18:1/14:0, d16:1/16:0)* (Lipid; Ceramides)
linoleoyl−arachidonoyl−glycerol (18:2/20:4) [1]* (Lipid; Diacylglycerol)
palmitoyl−linoleoyl−glycerol (16:0/18:2) [1]* (Lipid; Diacylglycerol)
stearoyl−linoleoyl−glycerol (18:0/18:2) [2]* (Lipid; Diacylglycerol)
myristoyl−linoleoyl−glycerol (14:0/18:2) [1]* (Lipid; Diacylglycerol)
myristoyl−linoleoyl−glycerol (14:0/18:2) [2]* (Lipid; Diacylglycerol)
oleoyl−linoleoyl−glycerol (18:1/18:2) [1] (Lipid; Diacylglycerol)
value
Visit
1 Wk
1 Mo
4 Mo
4.5 Mo
6 Mo
9 Mo
12 Mo
15 Mo
18 Mo

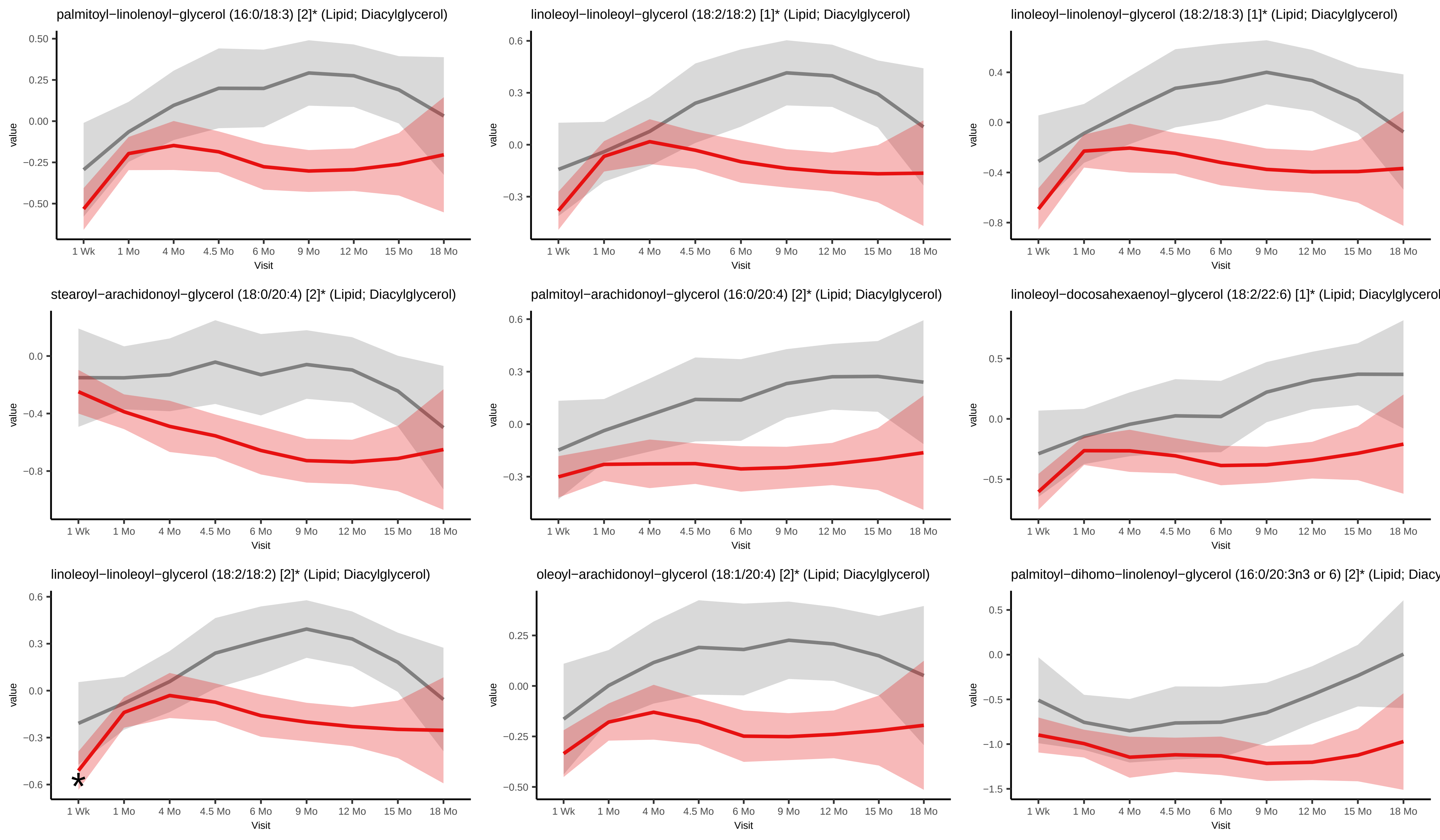
palmitoyl−linolenoyl−glycerol (16:0/18:3) [2]* (Lipid; Diacylglycerol)
0.50
0.25
0.00
−0.25
−0.50
value
1 Wk
1 Mo
4 Mo
4.5 Mo
6 Mo
9 Mo
12 Mo
15 Mo
18 Mo
Visit
linoleoyl−linoleoyl−glycerol (18:2/18:2) [1]* (Lipid; Diacylglycerol)
0.6
0.3
0.0
−0.3
value
Visit
linoleoyl−linolenoyl−glycerol (18:2/18:3) [1]* (Lipid; Diacylglycerol)
0.4
0.0
−0.4
−0.8
value
Visit
stearoyl−arachidonoyl−glycerol (18:0/20:4) [2]* (Lipid; Diacylglycerol)
0.0
−0.4
−0.8
value
Visit
palmitoyl−arachidonoyl−glycerol (16:0/20:4) [2]* (Lipid; Diacylglycerol)
0.6
0.3
0.0
−0.3
value
Visit
linoleoyl−docosahexaenoyl−glycerol (18:2/22:6) [1]* (Lipid; Diacylglycerol
0.5
0.0
−0.5
value
Visit
linoleoyl−linoleoyl−glycerol (18:2/18:2) [2]* (Lipid; Diacylglycerol)
0.6
0.3
0.0
−0.3
−0.6
value
*
Visit
oleoyl−arachidonoyl−glycerol (18:1/20:4) [2]* (Lipid; Diacylglycerol)
0.25
0.00
−0.25
−0.50
value
Visit
palmitoyl−dihomo−linolenoyl−glycerol (16:0/20:3n3 or 6) [2]* (Lipid; Diacy
0.5
0.0
−0.5
−1.0
−1.5
value
Visit

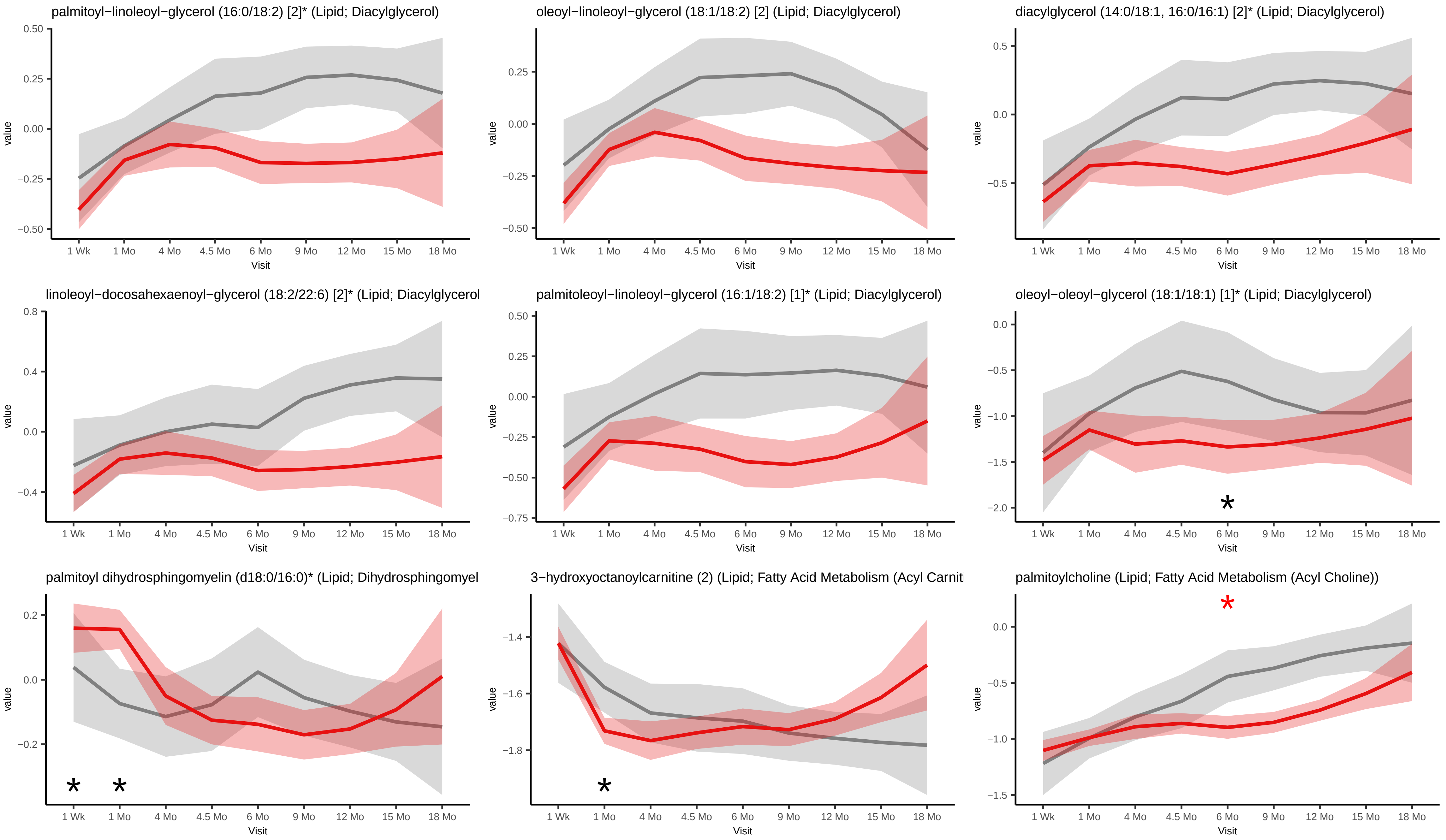
palmitoyl−linoleoyl−glycerol (16:0/18:2) [2]* (Lipid; Diacylglycerol)
oleoyl−linoleoyl−glycerol (18:1/18:2) [2] (Lipid; Diacylglycerol)
diacylglycerol (14:0/18:1, 16:0/16:1) [2]* (Lipid; Diacylglycerol)
linoleoyl−docosahexaenoyl−glycerol (18:2/22:6) [2]* (Lipid; Diacylglycerol
palmitoleoyl−linoleoyl−glycerol (16:1/18:2) [1]* (Lipid; Diacylglycerol)
oleoyl−oleoyl−glycerol (18:1/18:1) [1]* (Lipid; Diacylglycerol)
palmitoyl dihydrosphingomyelin (d18:0/16:0)* (Lipid; Dihydrosphingomyel
3−hydroxyoctanoylcarnitine (2) (Lipid; Fatty Acid Metabolism (Acyl Carniti
palmitoylcholine (Lipid; Fatty Acid Metabolism (Acyl Choline))
value
Visit
1 Wk
1 Mo
4 Mo
4.5 Mo
6 Mo
9 Mo
12 Mo
15 Mo
18 Mo
*

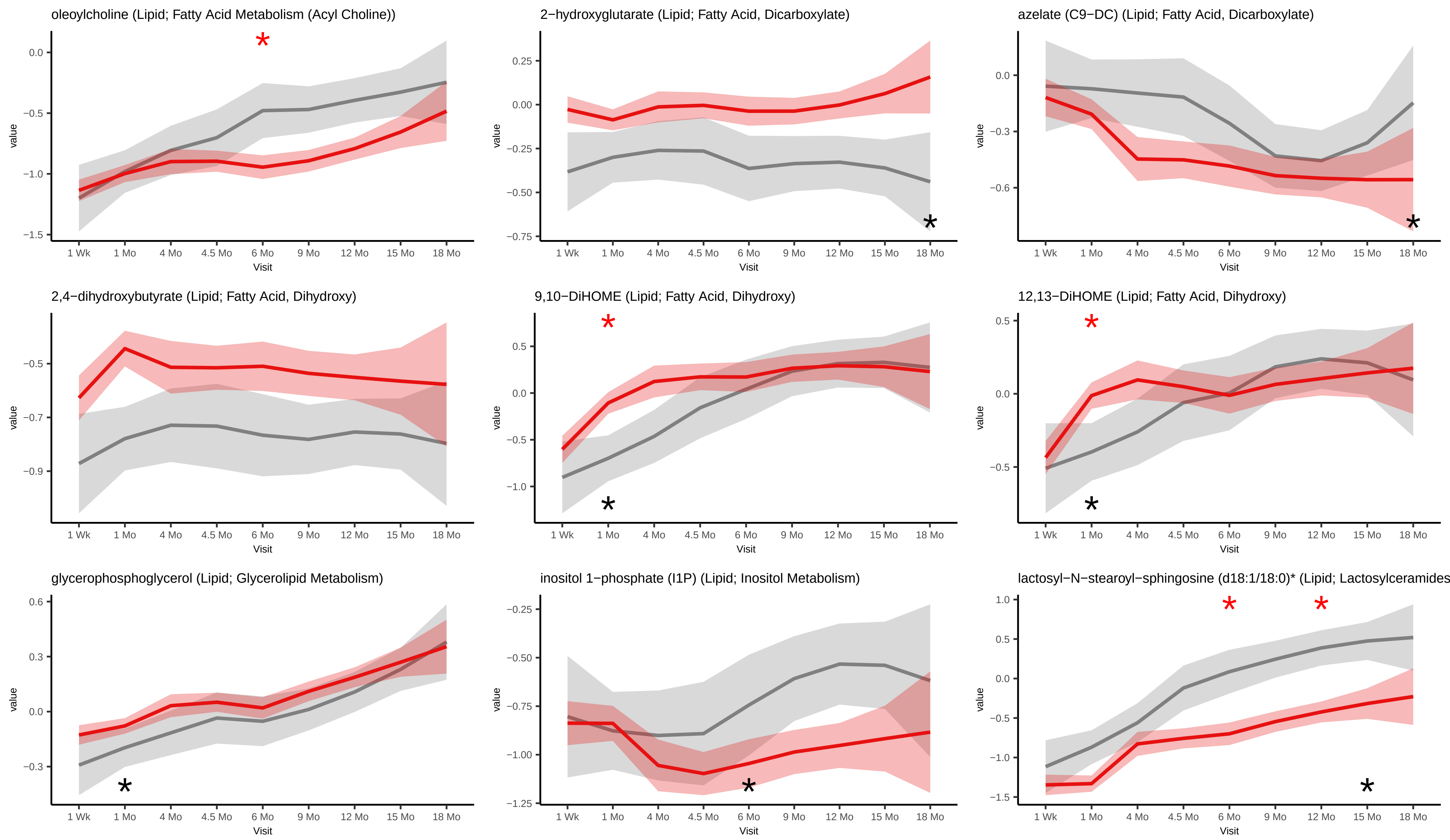

oleoylcholine (Lipid; Fatty Acid Metabolism (Acyl Choline))
2−hydroxyglutarate (Lipid; Fatty Acid, Dicarboxylate)
azelate (C9−DC) (Lipid; Fatty Acid, Dicarboxylate)
2,4−dihydroxybutyrate (Lipid; Fatty Acid, Dihydroxy)
9,10−DiHOME (Lipid; Fatty Acid, Dihydroxy)
12,13−DiHOME (Lipid; Fatty Acid, Dihydroxy)
glycerophosphoglycerol (Lipid; Glycerolipid Metabolism)
inositol 1−phosphate (I1P) (Lipid; Inositol Metabolism)
lactosyl−N−stearoyl−sphingosine (d18:1/18:0)* (Lipid; Lactosylceramides
value
Visit
1 Wk
1 Mo
4 Mo
4.5 Mo
6 Mo
9 Mo
12 Mo
15 Mo
18 Mo

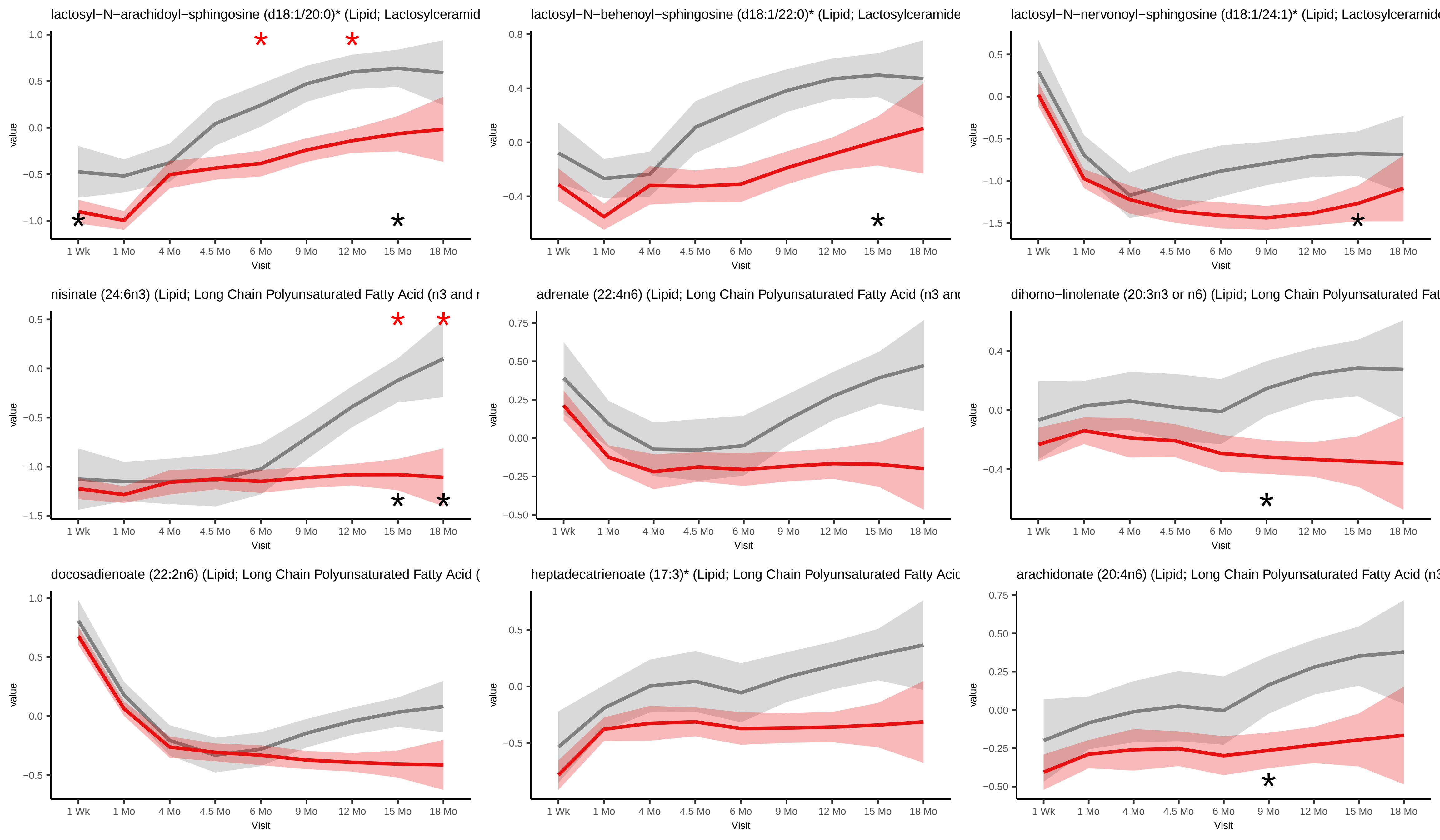
lactosyl−N−arachidoyl−sphingosine (d18:1/20:0)* (Lipid; Lactosylceramid
lactosyl−N−behenoyl−sphingosine (d18:1/22:0)* (Lipid; Lactosylceramide
lactosyl−N−nervonoyl−sphingosine (d18:1/24:1)* (Lipid; Lactosylceramid
nisinate (24:6n3) (Lipid; Long Chain Polyunsaturated Fatty Acid (n3 and r
adrenate (22:4n6) (Lipid; Long Chain Polyunsaturated Fatty Acid (n3 and
dihomo−linolenate (20:3n3 or n6) (Lipid; Long Chain Polyunsaturated Fat
docosadienoate (22:2n6) (Lipid; Long Chain Polyunsaturated Fatty Acid (
heptadecatrienoate (17:3)* (Lipid; Long Chain Polyunsaturated Fatty Acid
arachidonate (20:4n6) (Lipid; Long Chain Polyunsaturated Fatty Acid (n
value
Visit
1 Wk
1 Mo
4 Mo
4.5 Mo
6 Mo
9 Mo
12 Mo
15 Mo
18 Mo

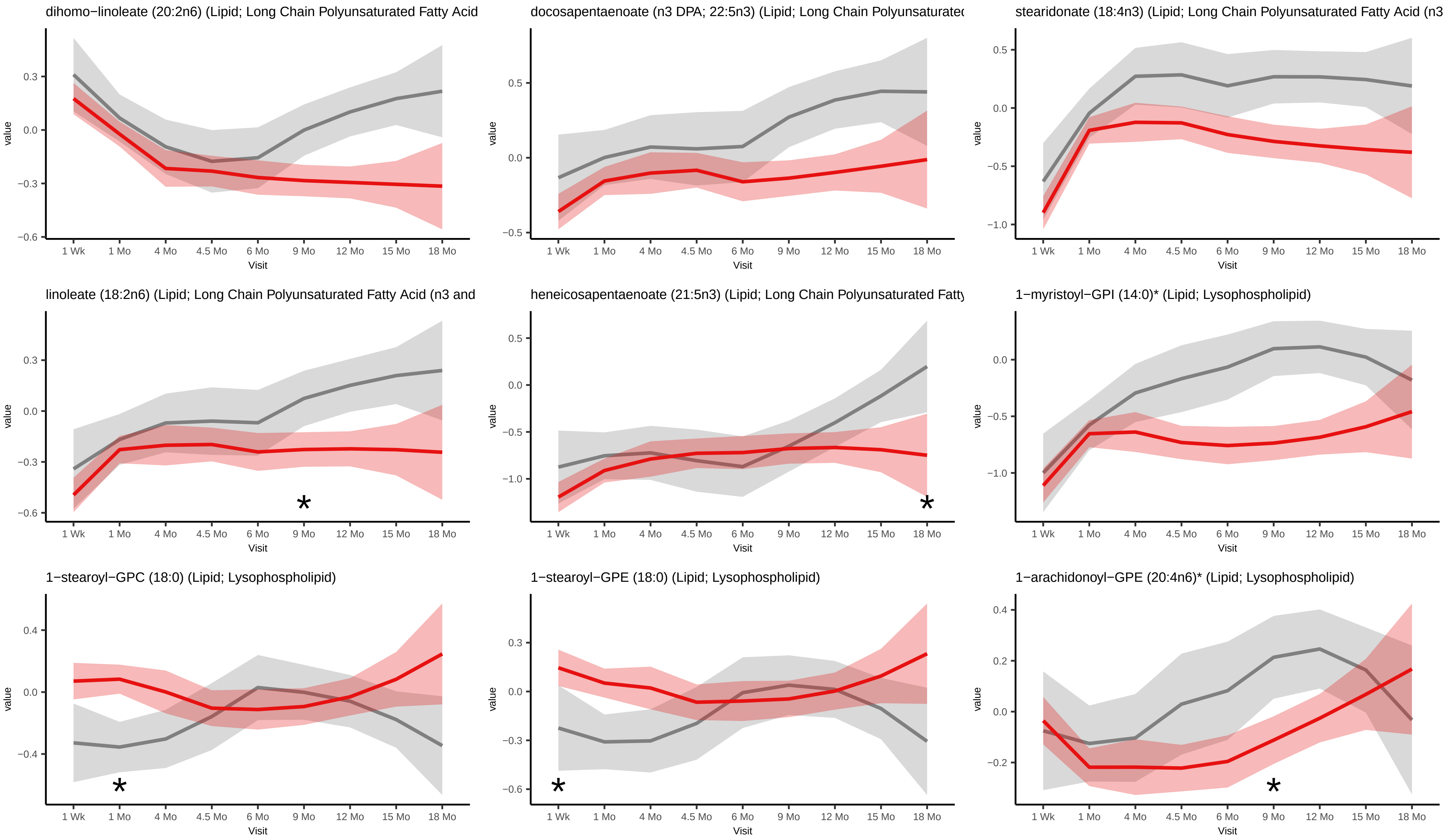

dihomo−linoleate (20:2n6) (Lipid; Long Chain Polyunsaturated Fatty Acid
docosapentaenoate (n3 DPA; 22:5n3) (Lipid; Long Chain Polyunsaturated
stearidonate (18:4n3) (Lipid; Long Chain Polyunsaturated Fatty Acid (n3
linoleate (18:2n6) (Lipid; Long Chain Polyunsaturated Fatty Acid (n3 and
heneicosapentaenoate (21:5n3) (Lipid; Long Chain Polyunsaturated Fatty
1−myristoyl−GPI (14:0)* (Lipid; Lysophospholipid)
1−stearoyl−GPC (18:0) (Lipid; Lysophospholipid)
1−stearoyl−GPE (18:0) (Lipid; Lysophospholipid)
1−arachidonoyl−GPE (20:4n6)* (Lipid; Lysophospholipid)
value
Visit
1 Wk
1 Mo
4 Mo
4.5 Mo
6 Mo
9 Mo
12 Mo
15 Mo
18 Mo
*

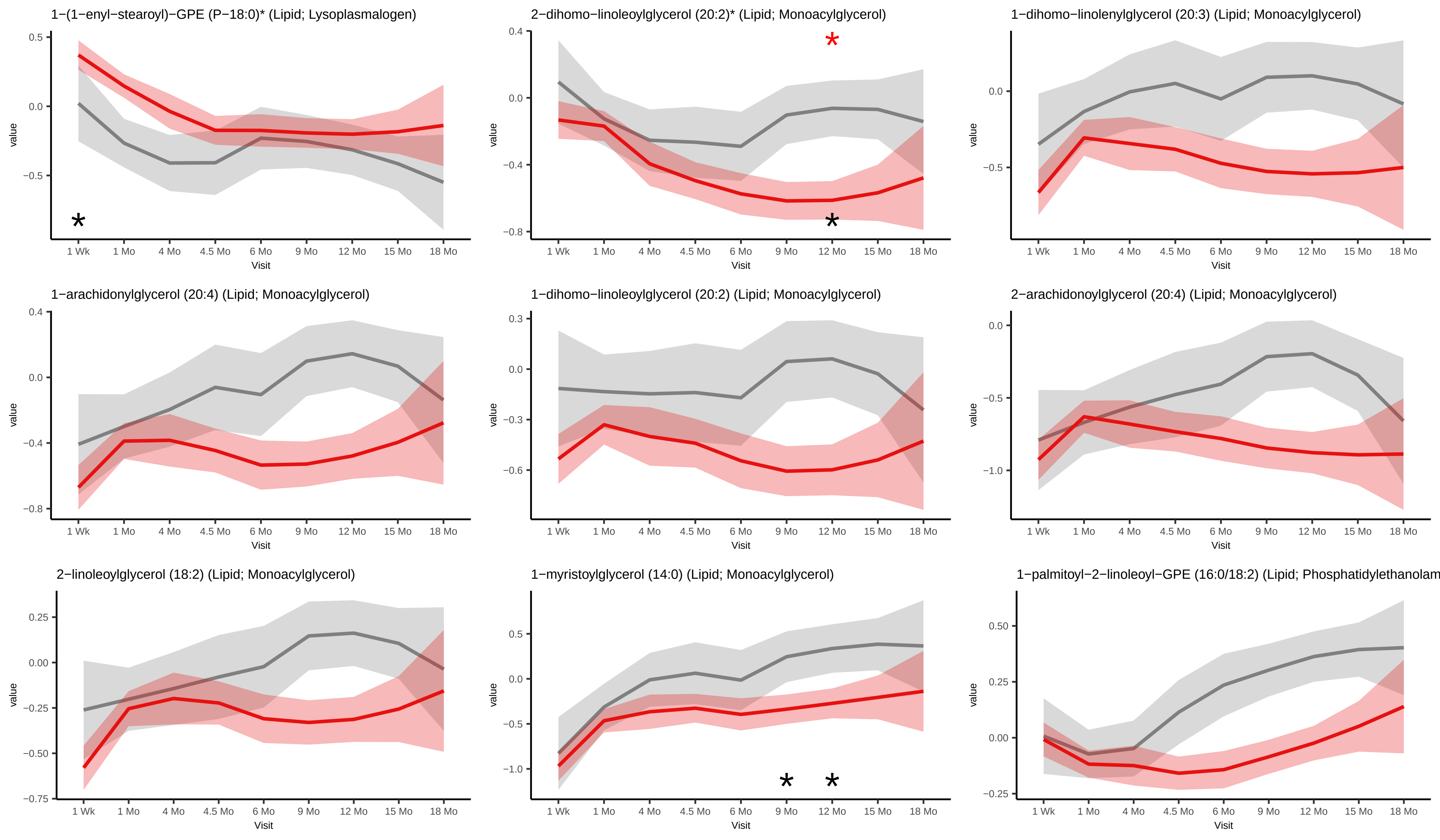
1−(1−enyl−stearoyl)−GPE (P−18:0)* (Lipid; Lysoplasmalogen)
2−dihomo−linoleoylglycerol (20:2)* (Lipid; Monoacylglycerol)
1−dihomo−linolenylglycerol (20:3) (Lipid; Monoacylglycerol)
1−arachidonylglycerol (20:4) (Lipid; Monoacylglycerol)
1−dihomo−linoleoylglycerol (20:2) (Lipid; Monoacylglycerol)
2−arachidonoylglycerol (20:4) (Lipid; Monoacylglycerol)
2−linoleoylglycerol (18:2) (Lipid; Monoacylglycerol)
1−myristoylglycerol (14:0) (Lipid; Monoacylglycerol)
1−palmitoyl−2−linoleoyl−GPE (16:0/18:2) (Lipid; Phosphatidylethanolam
value
Visit
1 Wk
1 Mo
4 Mo
4.5 Mo
6 Mo
9 Mo
12 Mo
15 Mo
18 Mo
*

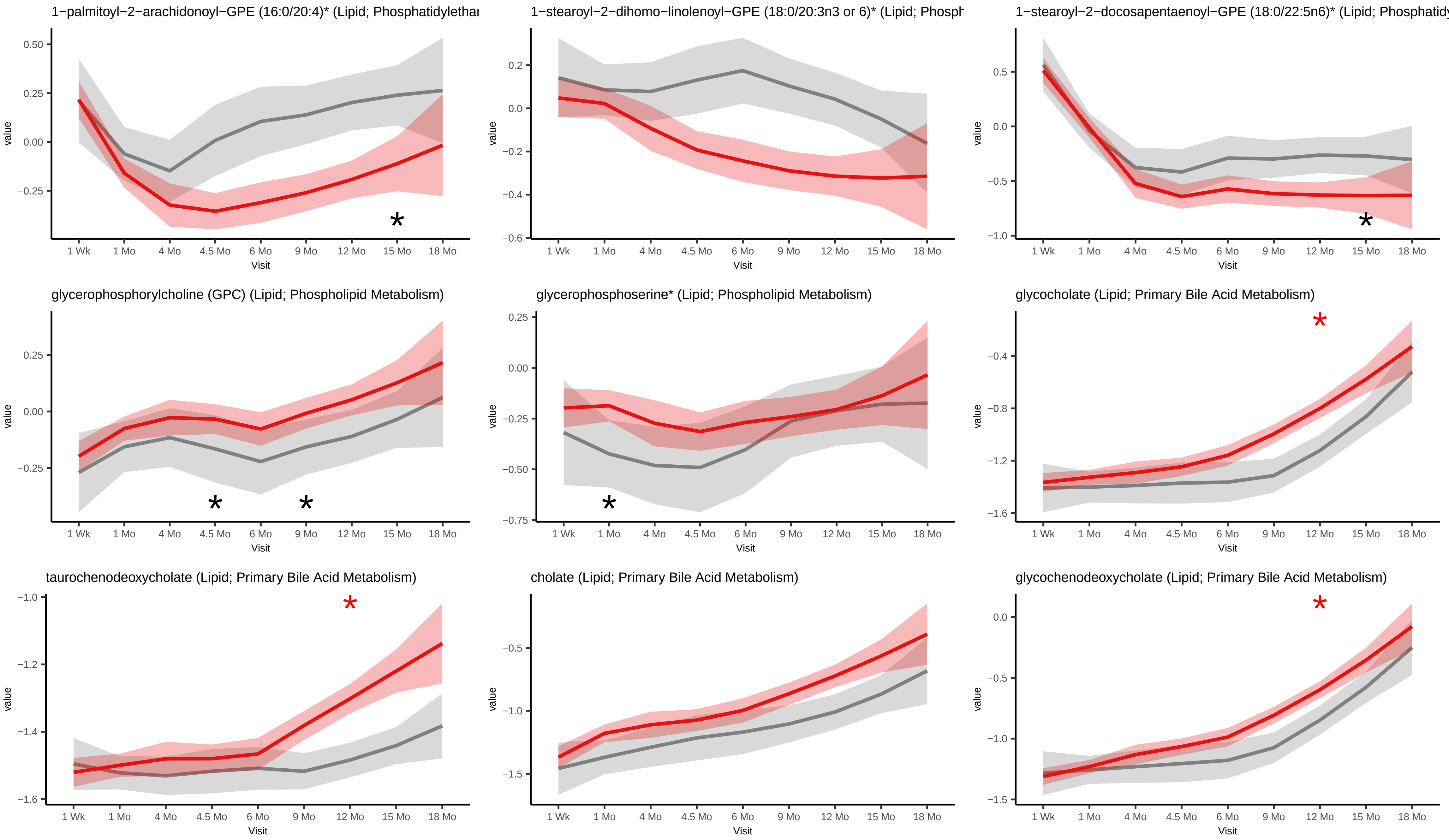
1−palmitoyl−2−arachidonoyl−GPE (16:0/20:4)* (Lipid; Phosphatidylethar
0.50
0.25
0.00
−0.25
value
*
1−stearoyl−2−dihomo−linolenoyl−GPE (18:0/20:3n3 or 6)* (Lipid; Phosph
0.2
0.0
−0.2
−0.4
−0.6
1−stearoyl−2−docosapentaenoyl−GPE (18:0/22:5n6)* (Lipid; Phosphatid
0.5
0.0
−0.5
−1.0
glycerophosphorylcholine (GPC) (Lipid; Phospholipid Metabolism)
glycerophosphoserine* (Lipid; Phospholipid Metabolism)
0.25
0.00
−0.25
−0.50
−0.75
glycocholate (Lipid; Primary Bile Acid Metabolism)
−0.4
−0.8
−1.2
−1.6
taurochenodeoxycholate (Lipid; Primary Bile Acid Metabolism)
−1.0
−1.2
−1.4
−1.6
cholate (Lipid; Primary Bile Acid Metabolism)
−0.5
−1.0
−1.5
glycochenodeoxycholate (Lipid; Primary Bile Acid Metabolism)
0.0
−0.5
−1.0
−1.5
1 Wk
1 Mo
4 Mo
4.5 Mo
6 Mo
9 Mo
12 Mo
15 Mo
18 Mo
Visit

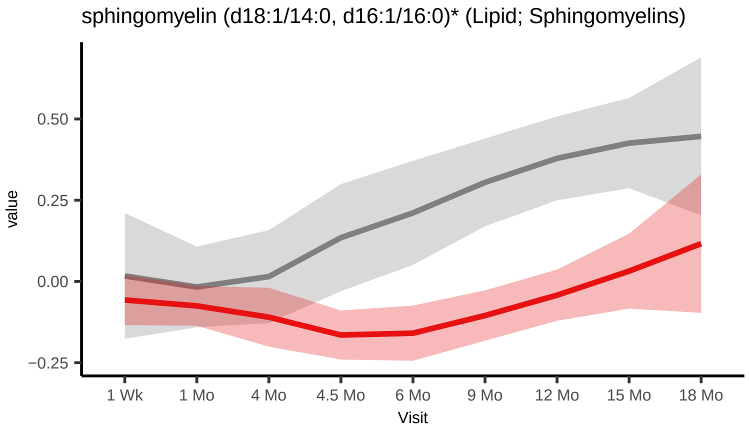
sphingomyelin (d18:1/14:0, d16:1/16:0)* (Lipid; Sphingomyelins)
value
0.50
0.25
0.00
−0.25
1 Wk
1 Mo
4 Mo
4.5 Mo
6 Mo
9 Mo
12 Mo
15 Mo
18 Mo
Visit

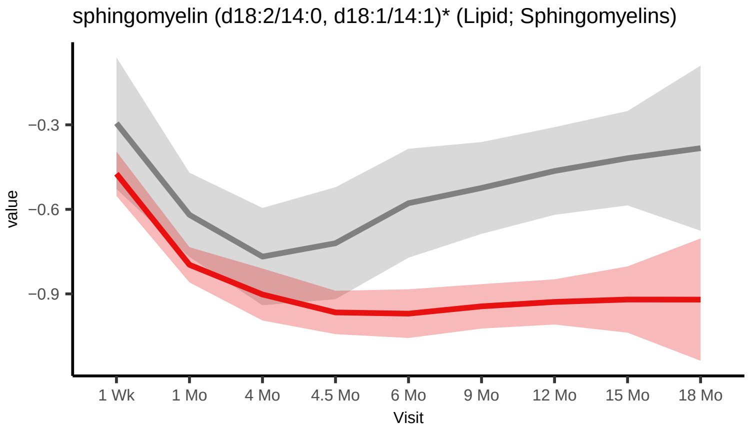
sphingomyelin (d18:2/14:0, d18:1/14:1)* (Lipid; Sphingomyelins)
value
−0.3
−0.6
−0.9
1 Wk
1 Mo
4 Mo
4.5 Mo
6 Mo
9 Mo
12 Mo
15 Mo
18 Mo
Visit

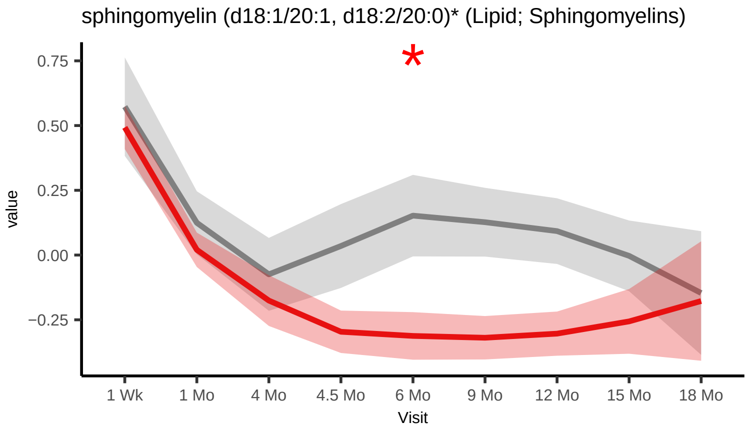
sphingomyelin (d18:1/20:1, d18:2/20:0)* (Lipid; Sphingomyelins)
*
value
0.75
0.50
0.25
0.00
−0.25
1 Wk
1 Mo
4 Mo
4.5 Mo
6 Mo
9 Mo
12 Mo
15 Mo
18 Mo
Visit

Nucleotide

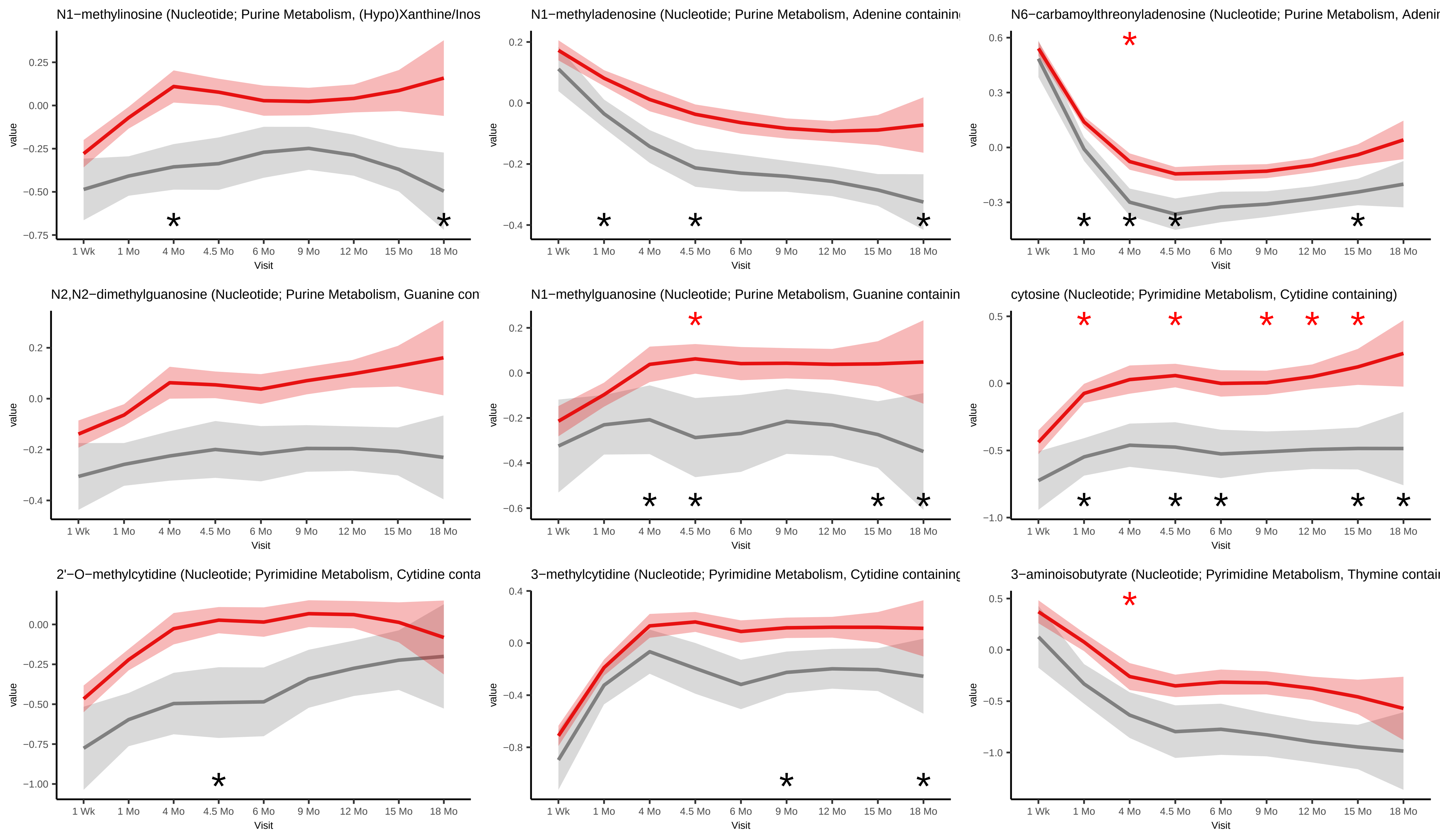

N1−methylinosine (Nucleotide; Purine Metabolism, (Hypo)Xanthine/Inos
N1−methyladenosine (Nucleotide; Purine Metabolism, Adenine containin
N6−carbamoylthreonyladenosine (Nucleotide; Purine Metabolism, Adenin
N2,N2−dimethylguanosine (Nucleotide; Purine Metabolism, Guanine con
N1−methylguanosine (Nucleotide; Purine Metabolism, Guanine containin
cytosine (Nucleotide; Pyrimidine Metabolism, Cytidine containing)
2'−O−methylcytidine (Nucleotide; Pyrimidine Metabolism, Cytidine conta
3−methylcytidine (Nucleotide; Pyrimidine Metabolism, Cytidine containing
3−aminoisobutyrate (Nucleotide; Pyrimidine Metabolism, Thymine contai
value
Visit
1 Wk
1 Mo
4 Mo
4.5 Mo
6 Mo
9 Mo
12 Mo
15 Mo
18 Mo

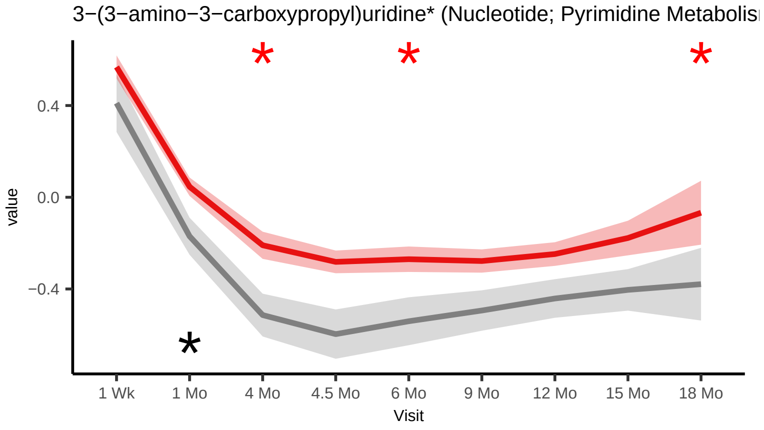
3−(3−amino−3−carboxypropyl)uridine* (Nucleotide; Pyrimidine Metabolis
0.4
0.0
−0.4
value
1 Wk
1 Mo
4 Mo
4.5 Mo
6 Mo
9 Mo
12 Mo
15 Mo
18 Mo
Visit

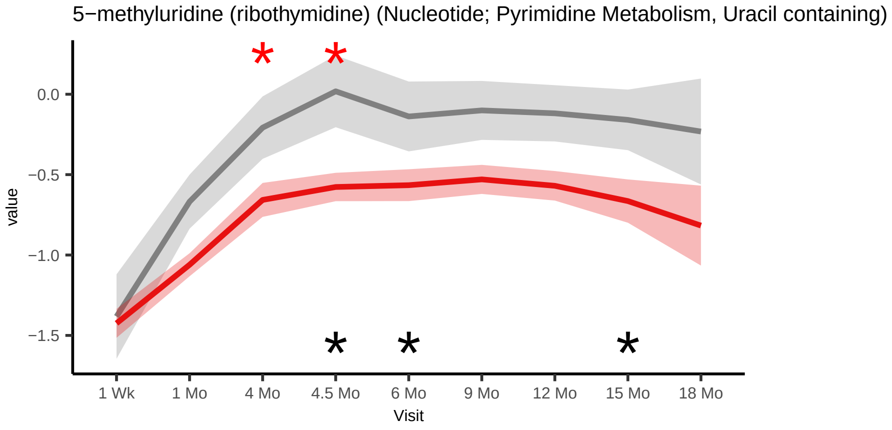
5−methyluridine (ribothymidine) (Nucleotide; Pyrimidine Metabolism, Uracil containing)
0.0
−0.5
−1.0
−1.5
value
1 Wk
1 Mo
4 Mo
4.5 Mo
6 Mo
9 Mo
12 Mo
15 Mo
18 Mo
Visit

Uncharacterized Compound

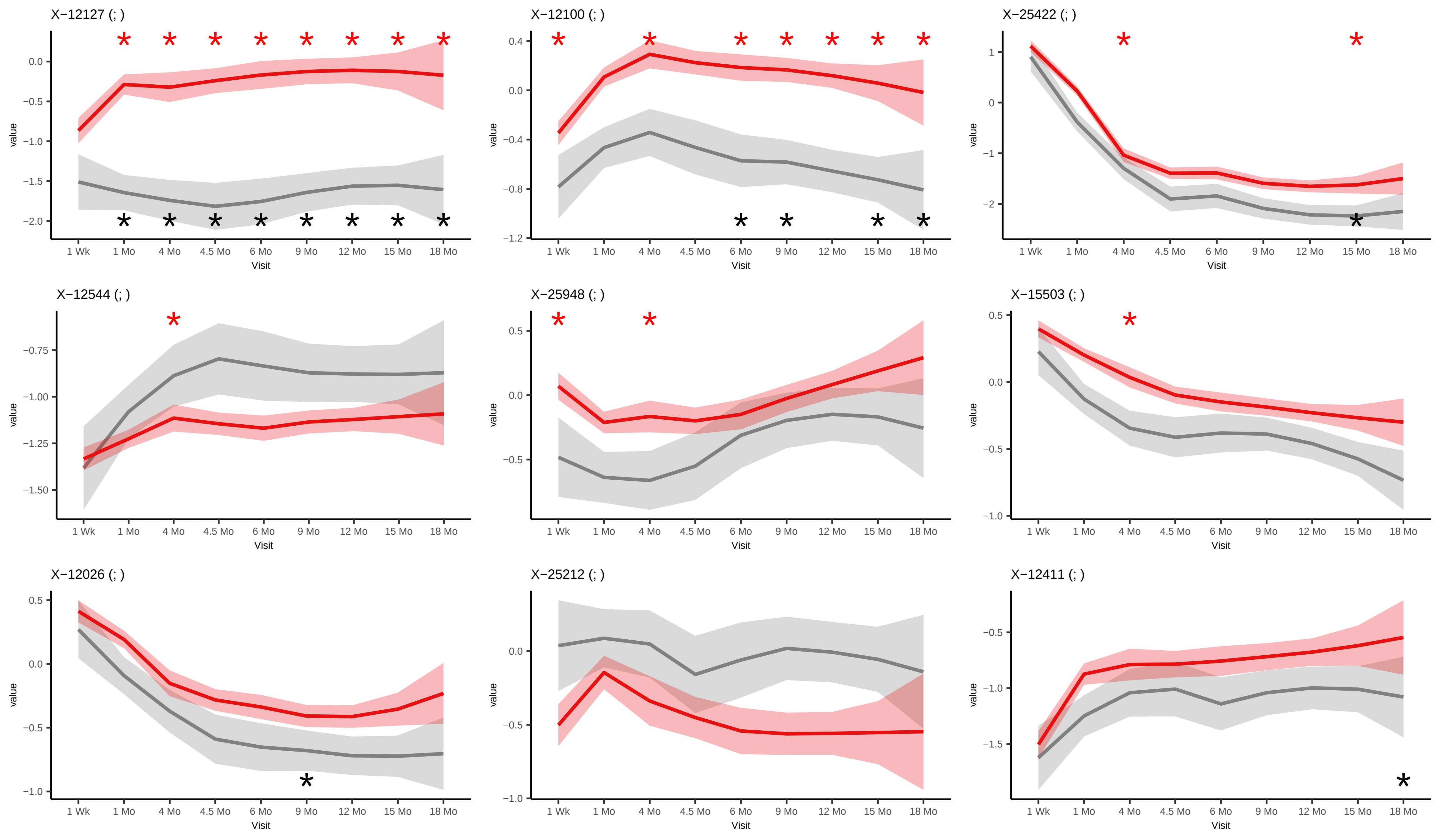
X−12127 (;)
0.0
−0.5
−1.0
−1.5
−2.0
value
1 Wk
1 Mo
4 Mo
4.5 Mo
6 Mo
9 Mo
12 Mo
15 Mo
18 Mo
Visit
X−12100 (;)
0.4
0.0
−0.4
−0.8
−1.2
X−25422 (;)
1
0
−1
−2
X−12544 (;)
−0.75
−1.00
−1.25
−1.50
X−25948 (;)
0.5
0.0
−0.5
X−15503 (;)
0.5
0.0
−0.5
−1.0
X−12026 (;)
0.5
0.0
−0.5
−1.0
X−25212 (;)
0.0
−0.5
−1.0
X−12411 (;)
−0.5
−1.0
−1.5

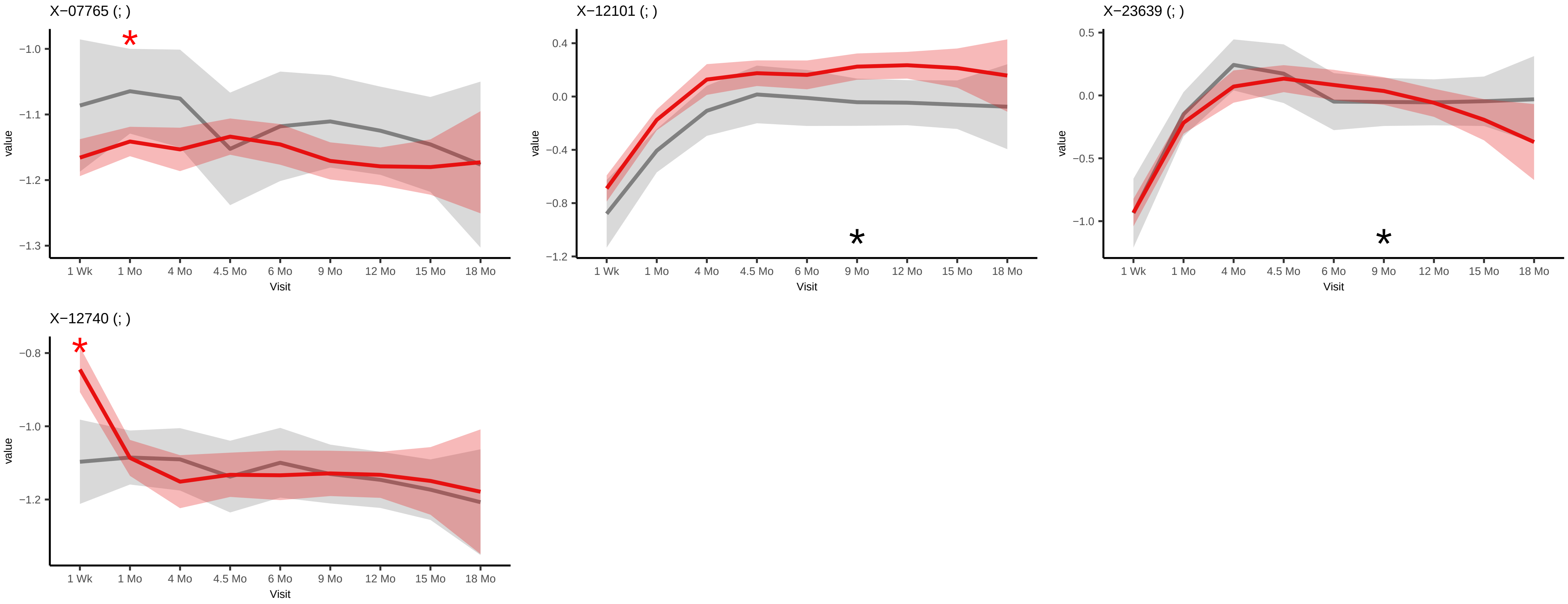
X−07765 (;)
X−12101 (;)
X−23639 (;)
X−12740 (;)
value
Visit
1 Wk
1 Mo
4 Mo
4.5 Mo
6 Mo
9 Mo
12 Mo
15 Mo
18 Mo
*

Xenobiotics

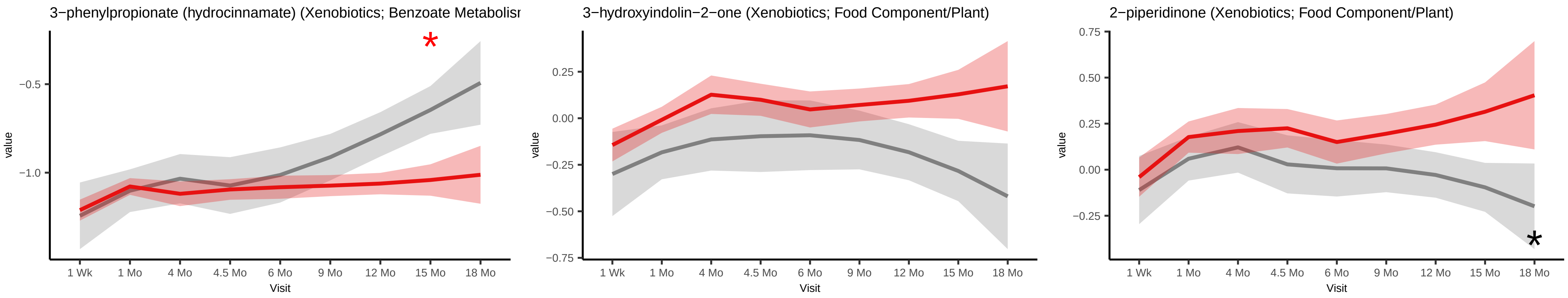
3−phenylpropionate (hydrocinnamate) (Xenobiotics; Benzoate Metabolisr
value
−0.5
−1.0
1 Wk
1 Mo
4 Mo
4.5 Mo
6 Mo
9 Mo
12 Mo
15 Mo
18 Mo
Visit
3−hydroxyindolin−2−one (Xenobiotics; Food Component/Plant)
value
0.25
0.00
−0.25
−0.50
−0.75
1 Wk
1 Mo
4 Mo
4.5 Mo
6 Mo
9 Mo
12 Mo
15 Mo
18 Mo
Visit
2−piperidinone (Xenobiotics; Food Component/Plant)
value
0.75
0.50
0.25
0.00
−0.25
1 Wk
1 Mo
4 Mo
4.5 Mo
6 Mo
9 Mo
12 Mo
15 Mo
18 Mo
Visit
